# Actuation‐Mediated Compression of a Mechanoresponsive Hydrogel by Soft Robotics to Control Release of Therapeutic Proteins

**DOI:** 10.1002/advs.202401744

**Published:** 2024-12-18

**Authors:** Eimear J. Wallace, Joanne O'Dwyer, Eimear B. Dolan, Liam P. Burke, Robert Wylie, Gabriella Bellavia, Stefania Straino, Francesca Cianfarani, Gabriella Ciotti, Simona Serini, Gabriella Calviello, Ellen T. Roche, Tapas Mitra, Garry P. Duffy

**Affiliations:** ^1^ Anatomy and Regenerative Medicine Institute (REMEDI) School of Medicine College of Medicine Nursing and Health Sciences University of Galway Galway H91 W2TY Ireland; ^2^ Explora‐Bioscience Srl G. Peroni 386 Rome 00131 Italy; ^3^ Pharmacology and Therapeutics School of Medicine College of Medicine Nursing and Health Sciences University of Galway Galway H91 W2TY Ireland; ^4^ CÚRAM SFI Research Centre for Medical Devices University of Galway Galway H91 W2TY Ireland; ^5^ Biomedical Engineering School of Engineering University of Galway Galway H91 HX31 Ireland; ^6^ Antimicrobial Resistance and Microbial Ecology Group School of Medicine, College of Medicine Nursing and Health Sciences University of Galway Galway H91 DK59 Ireland; ^7^ Centre for One Health Ryan Institute University of Galway Galway H91 DK59 Ireland; ^8^ Department of Translational Medicine and Surgery Section of General Pathology, Faculty of Medicine and Surgery Università Cattolica del Sacro Cuore Largo F. Vito Rome 1‐00168 Italy; ^9^ Institute for Medical Engineering and Science Massachusetts Institute of Technology Cambridge MA 01239 USA; ^10^ Harvard‐MIT Program in Health Sciences and Technology Cambridge MA 02139 USA; ^11^ Department of Mechanical Engineering Massachusetts Institute of Technology Cambridge MA 02139 USA; ^12^ SFI Centre for Advanced Materials and BioEngineering Research Centre (AMBER) Trinity College Dublin Dublin D02 W9K7 Ireland

**Keywords:** actuation, controlled drug delivery, hydrogels, soft robotics, therapeutic proteins

## Abstract

Therapeutic proteins, the fastest growing class of pharmaceuticals, are subject to rapid proteolytic degradation in vivo, rendering them inactive. Sophisticated drug delivery systems that maintain protein stability, prolong therapeutic effects, and reduce administration frequency are urgently required. Herein, a mechanoresponsive hydrogel is developed contained within a soft robotic drug delivery (SRDD) device. In a step‐change from previously reported systems, pneumatic actuation of this system releases the cationic therapeutic protein Vascular Endothelial Growth Factor (VEGF) in a bioactive form which is required for therapeutic angiogenesis, the growth of new blood vessels, in numerous clinical conditions. The ability of the SRDD device to release bioactive VEGF in a spatiotemporal manner from the hydrogel is tested in diabetic rats – a model in which angiogenesis is difficult to stimulate. Daily actuation of the SRDD device in the diabetic rat model significantly increased cluster of differentiation 31+ (CD31+) blood vessel number (p = 0.0335) and the diameter of alpha‐smooth muscle actin+ (α‐SMA+) blood vessels (p = 0.0025) compared to passive release of VEGF from non‐actuated devices. The SRDD device combined with the mechanoresponsive hydrogel offers the potential to deliver an array of bioactive therapeutics in a spatiotemporal manner to mimic their natural release in vivo.

## Graphical abstract

This work demonstrates the development of a mechanoresponsive hydrogel material that is incorporated into a pneumatically actuated soft robotic drug delivery device. The combined system can provide controlled protein delivery locally, as demonstrated using the therapeutic protein vascular endothelial growth factor.

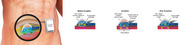

## 1 Introduction

Advanced drug delivery systems are devices or formulations developed to deliver therapeutics to a target site and minimize off‐target effects.^[^ ^1^, ^2^ ^]^ The therapeutic armamentarium has expanded to include small molecules, proteins, and peptides. The array of therapeutic molecules available means that traditional drug delivery systems, e.g., tablets and capsules are no longer sufficient, particularly for protein‐based therapeutics. Therapeutic proteins have become the fastest‐growing class of pharmaceuticals, representing 20% of the total number of drugs in development.^[^ ^3^, ^4^, ^5^, ^6^ ^]^ The global therapeutic protein market, with 239 therapeutic proteins approved by the Food and Drug Administration,^[^ ^7^ ^]^ was valued at $283.64 billion in 2020 and is expected to increase to $566.66 billion by 2030.^[^ ^8^ ^]^ Many therapeutic proteins are cationic^[^ ^9^, ^10^, ^11^, ^12^, ^13^, ^14^, ^15^ ^]^ and require spatiotemporal delivery to avoid proteolytic degradation in vivo.^[^ ^16^, ^17^ ^]^ Controlled drug delivery systems are being designed and developed to achieve this spatiotemporal delivery to increase the bioavailability of a therapeutic protein while also being non‐toxic and non‐immunogenic.^[^ ^1^, ^18^ ^]^ Controlled drug delivery systems have progressed over the last 50 years from first‐generation approaches depending on diffusion‐, osmosis‐, dissolution‐, and ion exchange‐based release mechanisms, e.g., once‐a‐day oral delivery systems and once‐a‐week transdermal patches, to second generation smart delivery systems, e.g. nano‐ and microparticles,^[^ ^19^, ^20^ ^]^ that release therapeutics in response to environmental factors such as temperature, pH, or glucose levels. However, less than 10 delivery systems are clinically available to deliver therapeutic proteins.^[^ ^21^, ^22^, ^23^, ^24^, ^25^, ^26^ ^]^ Several types of nanoparticles, microparticles, and hydrogels have been explored as advanced drug delivery systems,^[^ ^27^ ^]^ but achieving an implantable drug delivery system that can mimic the natural release mechanism of therapeutic proteins in the body such as the spatiotemporal release of growth factors remains a challenge. We are pursuing an alternative approach to nano‐ and microparticles as our goal is to develop a soft robotic drug delivery device which can control release rates and patterns for therapeutic proteins.

Soft robotic technologies made from materials that are soft in respect to their intended environment have become very popular in medical applications due to their inherent flexibility, conformability, compliance, and ability to achieve biomimetic motion.^[^ ^28^, ^29^, ^30^, ^31^ ^]^ Soft robotics are now being explored as devices that can be implanted in the body to mechanically interact with tissues and regulate biological function.^[^ ^28^, ^32^ ^]^ For example, Yim and Sitti developed a magnetically actuated soft capsule endoscope that changes to a spherical shape when magnetic actuation is externally applied to passively release drug in the gastrointestinal tract. The magnetically actuated soft capsule endoscope returns to its original cylindrical shape when all the drug is released so that the device can be expelled from the body. This approach should allow localized drug delivery once assessed in vivo but will lack tunable drug release rates.^[^ ^33^, ^34^ ^]^ Joyee and Pan also utilize an external magnetic field to control drug release from their multi‐material soft robot in human stomach models. The external magnetic field guides the multi‐material soft robot to the intended target site and also causes a downward deflection of the lower region of the drug reservoir to prevent drug release through the aperture on the upper region of the drug reservoir. Once the magnetic field is removed, the deflection of the bottom region of the drug reservoir is restored to its normal position forcing the drug up through the aperture. Drug release time and rate can be controlled by on‐off duration and strength of the applied external magnetic field.^[^ ^35^ ^]^ Similarly, Mendez et al. developed a hybrid hydrogel actuator that releases a positively charged drug in a spatiotemporal manner when pneumatically actuated.^[^ ^36^ ^]^ Despite demonstrating that soft robotic systems are implantable, and their actuations offer the potential to improve and control drug release rates locally, pneumatically actuated soft robotics have not yet been shown to release therapeutic proteins in a controlled spatiotemporal manner.

**Figure 1:**
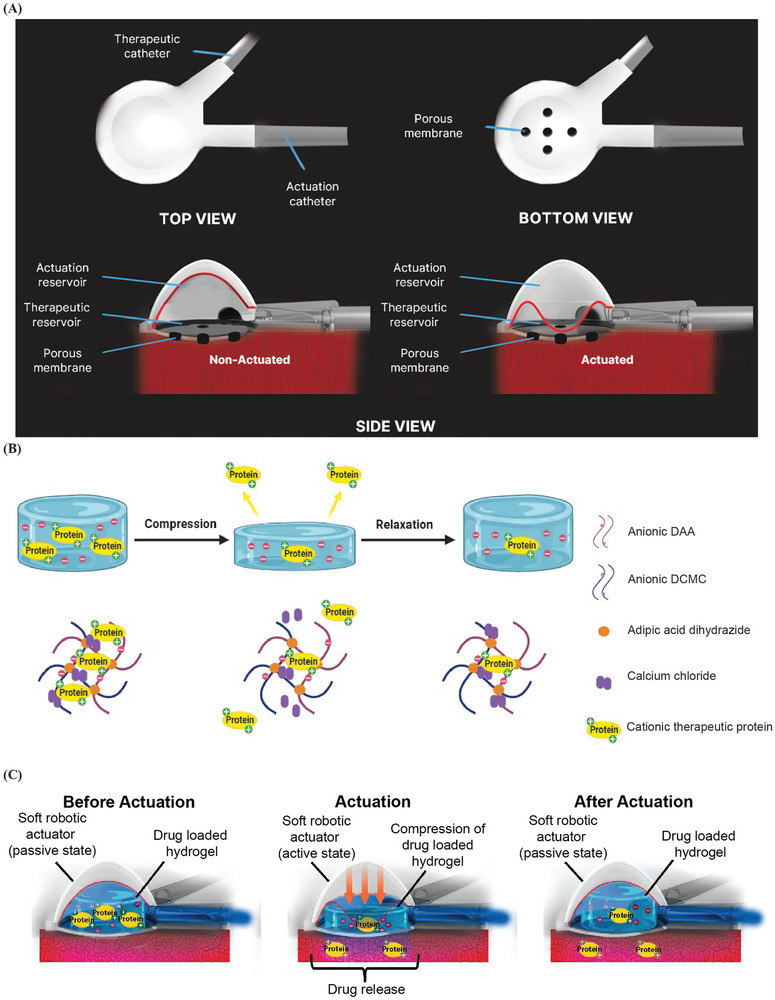
An overview of the mechanoresponsive alginic acid‐carboxymethylcellulose hydrogel and SRDD device. A) Schematic of SRDD device. B) Mechanoresponsive AA‐CMC hydrogel electrostatically sequestering a cationic protein. C) SRDD device loaded with AA‐CMC hydrogel electrostatically sequestering a cationic protein. Actuation of SRDD device compresses hydrogel to release protein locally in a controlled manner through the porous membrane of the SRDD device. DAA, dialdehyde alginic acid; DCMC, dialdehyde carboxymethylcellulose; SRDD, soft robotic drug delivery.

We have developed an implantable pneumatically actuatable soft robotic drug delivery (SRDD) device (**Figure** **1**A) and formulated a mechanoresponsive hydrogel that, when coupled, can release therapeutic proteins in a controlled spatiotemporal manner. Mechanoresponsive hydrogels respond to mechanical stimuli by changing their shape, volume, stiffness, permeability to release encapsulated substances.^[^ ^37^, ^38^ ^]^ The therapeutic reservoir of the SRDD device is loaded with an anionic alginic acid (AA)‐carboxymethylcellulose (CMC) hydrogel that can electrostatically interact with a cationic protein, e.g., vascular endothelial growth factor (VEGF), to sequester it within the reservoir and protect it from enzymatic degradation by circulating proteases prior to its intended release in vivo. VEGF is an ideal model therapeutic protein to assess the ability of the SRDD system to release a therapeutic in a temporal, localized manner while maintaining its bioactivity, as bioactivity can be measured by quantifying local blood vessel formation (angiogenesis).^[^ ^39^, ^40^ ^]^ Clinically, VEGF can treat a variety of medical conditions caused by ischemia. However, if VEGF is not delivered locally in a spatiotemporal manner it can result in adverse off‐target effects, e.g., aberrant blood vessel formation, pathological vascularization of dormant tumors, worsening of diabetic retinopathy.^[^ ^41^, ^42^ ^]^ The advantage of using VEGF as a model therapeutic is that its bioactivity can be determined by quantifying the amount of blood vessel formation, off‐target effects are well defined and can be assessed and advanced drug delivery systems for VEGF are clinically desirable.^[^ ^39^, ^40^ ^]^ The AA‐CMC hydrogel is mechanoresponsive (Figure 1B) and so when the SRDD device is pneumatically actuated it compresses the hydrogel to release the sequestered VEGF in a spatiotemporal manner (Figure 1C). Similar to the hybrid hydrogel actuator developed by Mendez et al.,^[^ ^36^ ^]^ the release rate and pattern from the SRDD system are tunable by modifying actuation parameters, i.e., pressure, ramp times, cycle number. The resultant actuation regime facilitates release of VEGF from the SRDD system as actuation compresses the therapeutic reservoir of the SRDD device, which compresses the AA‐CMC hydrogel, disrupting the electrostatic interactions between the hydrogel polymers and VEGF.^[^ ^43^, ^44^, ^45^, ^46^, ^47^, ^48^ ^]^ Subsequent actuations facilitate further release of VEGF to achieve repeated drug delivery. In this paper, we demonstrate that daily actuation of the SRDD system facilitates spatiotemporal delivery of VEGF for 7 days in rodent models to stimulate therapeutic angiogenesis locally at the implant site proving that the bioactivity of VEGF is maintained with this delivery platform.

## 2 Results

### 2.1 Structural and Physiochemical Analysis of AA‐CMC Hydrogel

**Figure 2:**
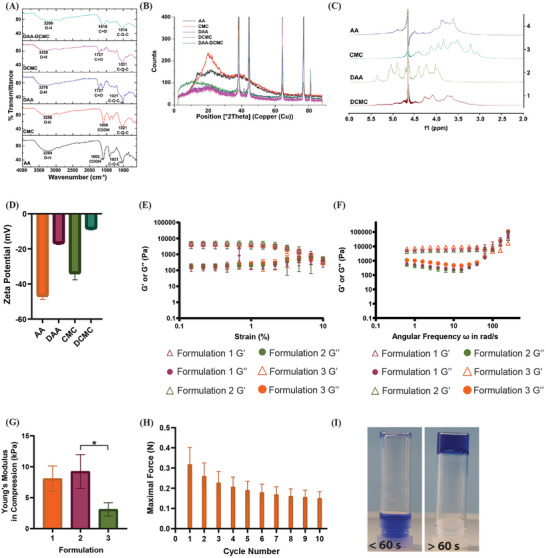
Physiochemical analysis of AA‐CMC hydrogel. A) Fourier transform infrared spectra of AA, CMC, DAA, and DCMC, B) X‐ray diffraction patterns of AA, CMC, DAA, and DCMC, C) nuclear magnetic resonance spectra of AA, CMC, DAA, and DCMC, D) zeta potential of AA, CMC, DAA, and DCMC, E) amplitude sweep of different formulations of AA‐CMC hydrogel, F) frequency sweep of different formulations of AA‐CMC hydrogel, G) Young's modulus calculated between 10 and 20% strain of different formulations of AA‐CMC hydrogel, H) maximum force required to produce 40% compression of AA‐CMC hydrogel formulation 2, I) gelation of AA‐CMC hydrogel (using methylene blue for visualization). *N* = 3 per group with data presented as means ± standard deviation, one‐way ANOVA, *p < 0.05. AA, alginic acid; CMC, carboxymethylcellulose; DAA, dialdehyde alginic acid; DCMC, dialdehyde carboxymethylcellulose; G’, storage modulus; G’’, loss modulus.

A significant challenge in achieving spatiotemporal drug release from a device is sequestering the drug within the device reservoir until release is desired. Our envisioned system required a hydrogel that could sequester a charged therapeutic protein and release a specific amount of the protein at a defined time, locally, in response to an external stimulus. Thus, we sought to develop a mechanoresponsive, anionic hydrogel that could sequester a cationic therapeutic protein and release it spatiotemporally in response to an applied mechanical force. The polymers required oxidation to allow formation of the AA‐CMC hydrogel and so Fourier Transform Infrared Spectroscopy was used to confirm the oxidation of AA and CMC with spectra for AA, CMC, dialdehyde alginic acid (DAA), and dialdehyde carboxymethylcellulose (DCMC) shown in **Figure** **2**A. The AA and CMC spectra had characteristic bands of polysaccharides at 1021‐1014 cm^−1^ (C‐O‐C stretching of the pyranose ring skeleton) with a band at 1600 cm^−1^ corresponding to the carboxyl group.^[^ ^49^ ^]^ For DAA and DCMC spectra, a new band located at 1727 cm^−1^ is attributed to the carbonyl group stretching of the aldehyde group confirming the oxidation of AA and CMC. Moreover, the bands at 1021‐1014 cm^−1^ are reduced in DAA and DCMC due to the introduction of the aldehyde groups. The band at 1727 cm^−1^ for DAA and DCMC disappeared in the AA‐CMC hydrogel with a new peak at 1610 cm^−1^, indicating the formation of a Schiff base between the amino groups of adipic acid dihydrazide (ADH) and the aldehyde functional group of DAA and DCMC. X‐ray diffractometer analysis was used to detect potential crystalline phases in hydrogels. As shown in Figure 2B, the X‐ray diffractometer patterns of AA and CMC both exhibited a broad peak centered at ~ 20° (2θ), suggesting the amorphous state of polymers. However, such peaks were absent in DAA and DCMC due to the aldehyde disruption of the C2 and C3 hydroxyl structure, further confirming the successful oxidation of the AA and CMC polymers. The nuclear magnetic resonance spectra obtained for AA, CMC, DAA and DCMC are shown in Figure 2C. The signals at the 4.5‐3 ppm for both AA and CMC represent the backbone of the alginate and cellulose carbohydrates (C‐H), which are chemically similar. Upon oxidation producing DAA and DCMC, the signals shift to a higher ppm, 5.5‐4 ppm, due to the introduction of aldehyde groups increasing the ease at which protons move in the presence of the applied magnetic field. The degree of oxidation within the final 10% w/v AA‐CMC hydrogel was confirmed as 40 ± 3.5% using aldehyde assays where the number of aldehydes within the hydrogel was determined.

Zeta potential measures the surface charge of substances and was performed to confirm the negative charge of the hydrogel polymers to ensure electrostatic interactions could form with a cationic protein. As shown in Figure 2D the zeta potential of DAA and DCMC was ‐16.90 ± 0.67 and ‐8.55 ± 0.63 mV, respectively. The mechanical properties of the AA‐CMC hydrogel were then investigated by rheology and compressive strength experiments. Strain sweeps were conducted to determine the response of the hydrogels to increasing strain and obtain their linear viscoelastic range. All formulations of the AA‐CMC hydrogel (**Table** **1**) had a constant storage modulus up to 10% strain, after which a reduction occurred (Figure 2E). Further, in frequency sweeps, all formulations of the AA‐CMC hydrogel remained intact at 0.1% strain and oscillations up to 100 Hz frequency, indicating the hybrid cross‐linking of the AA‐CMC hydrogel should withstand the strain likely to be applied by actuation of the SRDD device. Rheology demonstrated that all formulations of AA‐CMC hydrogels were predominantly elastic at angular frequencies > 100 rad/s as storage moduli (G’) exceeded loss moduli (G’’) as shown in Figure 2F with no significant differences between the moduli of the different formulations (*p* = 0.9917). The mechanical properties of the AA‐CMC hydrogel were further investigated by mechanical compression tests to determine the optimal formulation required to obtain a hydrogel with high mechanical strength, i.e., a robust hydrogel to withstand mechanical compression during actuation of the SRDD device. The amount of ADH differed between the formulations of the AA‐CMC hydrogels in Table 1. Increasing the amount of ADH in the formulation improved mechanical stability and reduced gelation time (Table 1). We hypothesize that this is due to an increased amount of covalent interactions within the hydrogel. Simultaneously, the amount of DI H_2_O was adjusted to ensure the total volume of each formulation remained constant. As demonstrated in Figure 2G the compressive modulus of the AA‐CMC hydrogel formulation 2 was the highest at 9.24 ± 2.72 kPa and so this formulation progressed to cyclic compression testing. The AA‐CMC hydrogel formulation 2 was subjected to cyclic compressions to determine its ability to recover from successive compressions. Despite a gradual non‐significant reduction in the maximal force required to compress the hydrogel with each successive compression, the hydrogel withstood all 10 cycles of compression, a cycle number to be used preclinically to deliver therapeutic protein doses, as shown in Figure 2H. Thus, demonstrating that the AA‐CMC hydrogel formulation 2 will remain intact in the presence of compressive forces when integrated with the SRDD device. An inverted tube test was carried out on the AA‐CMC formulation 2 to determine its time to gelation to ensure the formulation will remain viscous for sufficient time to facilitate delivery via the intended delivery route of injection through a 23 G needle and then through the therapeutic catheter of the SRDD device with an inner diameter of 0.58 mm. This formulation remained viscous for ~ 60 s with gelation thereafter as depicted in Figure 2I. The AA‐CMC formulation 2 was injected through a 23 G needle and then through the therapeutic catheter of the SRDD device, demonstrating that it could be injected by a human syringe operator and form a hydrogel within the therapeutic reservoir of the SRDD device (Movie S1, Supporting Information).

**Table 1:** AA‐CMC hydrogel formulation for characterization studies.

AA‐CMC Formulation	Glass vial 1		Glass vial 2			Gelation time
	DAA	DCMC	ADH	CaCl_2_	Deionized water	
	10% w/v dissolved in deionized water	10% w/v dissolved in deionized water	0.5 m dissolved in deionized water	0.2 m dissolved in deionized water		
1	50 µL	50 µL	60 µL	40 µL	0 µL	< 60 s
2	50 µL	50 µL	50 µL	40 µL	10 µL	60–120 s
3	50 µL	50 µL	30 µL	40 µL	40 µL	> 120 s

ADH adipic acid dihydrazide; CaCl_2_, calcium chloride; DAA, dialdehyde alginic acid; DCMC, dialdehyde carboxymethylcellulose.

### 2.2 Design and Fabrication of a SRDD Device

Previous studies have shown a dynamic soft reservoir device utilized pneumatic actuation to attenuate the foreign body response to implanted devices short‐term (2 weeks).^[^ ^28^ ^]^ The next generation device, soft transport augmenting reservoir, also utilized pneumatic actuation and mitigated the foreign body response to implanted devices long‐term (8 weeks) while also maintaining drug delivery during this period.^[^ ^50^ ^]^ These previous studies demonstrated the potential to employ soft robotics to reduce the development of fibrous tissue around implanted devices and the ability to deliver a drug analogue (Genhance 750) in an on‐demand, actuation‐mediated manner. Building on those previous studies, the SRDD device, depicted in Figure 1A, was developed with a porous membrane and coupled with the mechanoresponsive AA‐CMC formulation 2 hydrogel to test the hypothesis that spatiotemporal delivery of a therapeutic can be achieved at implant sites by actuation of the SRDD device. The SRDD device was composed of thermoplastic polyurethane that has an elastic modulus of ~ 15 MPa, which is similar to the extracellular matrix.^[^ ^51^ ^]^ Further, thermoplastic polyurethane has been used in commercially available medical devices, making it a good choice for potential future translation.^[^ ^52^, ^53^, ^54^ ^]^ The therapeutic reservoir of the SRDD device, preloaded with the AA‐CMC hydrogel, has a membrane with 5 laser‐cut 0.3048 mm diameter pores that are in direct contact with underlying tissue. The actuation catheter is connected to a transcutaneous port to facilitate pressurization of the implanted actuation reservoir of the SRDD device to deflect the therapeutic membrane downwards and compress the AA‐CMC formulation 2 hydrogel (Movie S2, Supporting Information). The SRDD device developed in this manuscript was scaled for implantation in rats but can be scaled‐up for large animal models or clinical translation by modifying the 3D printed molds, thermoforming, and heat‐sealing process, and tuning the actuation regime.

### 2.3 Customizable Actuation Regime Offers Tunable Release Rates of Drug from SRDD System

**Figure 3:**
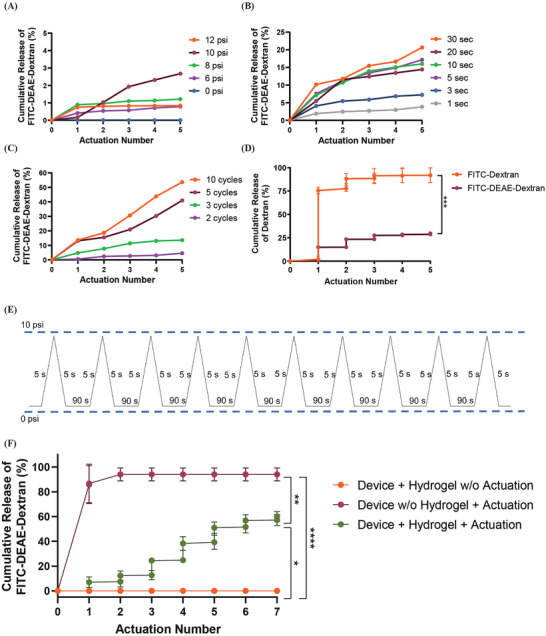
Optimization of actuation regime parameters demonstrates that FITC‐DEAE‐Dextran release from SRDD system is tunable. Optimization of actuation regime parameters A) pressure, B) ramp, and C) cycle number. *N* = 3 per group with data presented as means only. D) Effect of charge on Dextran release. *N* = 3 per group with data presented as means ± standard deviation, One‐way ANOVA, ***p < 0.0005. E) Optimized actuation profile of 10 psi, 5 s triangle ramp, 10 cycles. F) Controlled release of FITC‐DEAE‐Dextran with the combination of SRDD device, AA‐CMC based hydrogel and actuation. *N* = 3 per group with data presented as means ± standard deviation. One‐way ANOVA, *p < 0.05, **p < 0.01, ***p < 0.005, ****p < 0.001. AA, alginic acid; CMC, carboxymethylcellulose; DEAE, Diethylaminoethyl; FITC, Fluorescein isothiocyanate; SRDD, soft robotic drug delivery.

As depicted in **Figure** **3**A, a range of actuation pressures were investigated with 6, 8, 10, and 12 psi releasing 0.78 ± 0.28%, 1.22 ± 0.45%, 2.68 ± 1.13%, and 0.84 ± 0.33% of the loaded Fluorescein isothiocyanate–Diethylaminoethyl–Dextran (FITC‐DEAE‐Dextran, positively charged Dextran), respectively. However, the highest pressure tested, 12 psi, irreversibly damaged the structure of the AA‐CMC formulation 2 hydrogel within the therapeutic reservoir of the SRDD device. Therefore, 10 psi was chosen as the optimal pressure for the actuation regime. A range of ramp times were then examined to try to increase cumulative release of FITC‐DEAE‐Dextran. Increasing ramp times from 1 – 30 s increased the cumulative release of FITC‐DEAE‐Dextran with 5 and 30 s releasing 17.17 ± 6.19% and 20.64 ± 7.13% of the loaded FITC‐DEAE‐Dextran respectively as shown in Figure 3B. A ramp time of 30 s released the largest cumulative amount of FITC‐DEAE‐Dextran but was not significantly greater (*p* = 0.6606) than that released by a 5 s ramp time. Further, a 30 s ramp time translates to a 60 s actuation time, while a ramp time of 5 s translates to a 10 s actuation time, i.e., 5 s ramp results in a shorter treatment delivery time and was subsequently selected as the optimal ramp time. With 10 psi and a 5 s ramp selected, a range of cycle numbers (2, 3, 5, and 10) were examined to investigate if this could increase FITC‐DEAE‐Dextran release. As shown in Figure 3C, actuations of 2, 3, and 5 cycles resulted in a cumulative release of 4.56 ± 1.69%, 13.58 ± 5.33%, and 40.98 ± 14.24% of the loaded FITC‐DEAE‐Dextran, respectively. The 10 cycles increased FITC‐DEAE‐Dextran cumulative release to 53.61 ± 19.90% and was chosen for incorporation into the optimized actuation regime (10 cycles of 10 psi, 10 s on and 90 s off). The AA‐CMC formulation 2 hydrogel was prepared with neutral Dextran (FITC‐Dextran), and positively charged Dextran (FITC‐DEAE‐Dextran) to determine the effect of charge on the release pattern of drug from the SRDD system. FITC‐Dextran (neutral) demonstrated a burst release with the first actuation of the SRDD device releasing 75.55 ± 33.31% of the total loaded drug as seen in Figure 3D, while just 14.81 ± 10.62% of the positively charged FITC‐DEAE‐Dextran was released. The cumulative release of FITC‐Dextran was 92.00 ± 35.53% which was significantly greater (*p* = 0.0053) than the cumulative release of FITC‐DEAE‐Dextran obtained at the end of the actuation regime (29.65 ± 10.93%). The optimized SRDD devices fabricated with 5 evenly distributed 0.3048 mm diameter, non‐rate limiting, laser‐cut pores were used from this point onwards to ensure uniform porosity. The optimized actuation regime shown in Figure 3E when combined with the SRDD devices with uniform porosity released 59.97 ± 5.74% of the total loaded FITC‐DEAE‐Dextran in a controlled manner over a period of 7 days as illustrated in Figure 3F. In addition, the therapeutic reservoir of the SRDD device was refilled with the AA‐CMC hydrogel after the 70 consecutive in vitro actuations (to recapitulate the animal studies discussed below) as shown in Movie S3 (Supporting Information).

### 2.4 Establishing a Diabetic Rat Model

We implanted SRDD devices loaded with AA‐CMC hydrogel with or without VEGF in healthy rats initially and observed VEGF released by actuation was bioactive and stimulated angiogenesis locally at implant sites compared to control devices without VEGF (Supporting Information). Diabetes has many associated co‐morbidities that involve ischemia (e.g., peripheral vascular disease), but therapeutic angiogenesis is more difficult to stimulate in diabetes.^[^ ^55^, ^56^, ^57^, ^58^ ^]^ An envisioned clinical translation of the SRDD system is to deliver cationic VEGF locally in a stimuli‐responsive spatiotemporal manner to pre‐vascularize an implant site prior to islet transplantation to improve islet viability. Thus, a type 1 phenotype diabetic model needed to be established to assess the therapeutic efficacy of the spatiotemporally delivered VEGF. A streptozotocin‐induced diabetic rat model was established by administering fasted female Sprague Dawley rats (aged 11 weeks, 250–310 g) 65 mg kg^−1^ Streptozotocin (STZ) intraperitoneally. Rats were provided access to 600 mL of 10% w/v sucrose solution for 48 h to reduce the risk of STZ‐induced hypoglycemia. Hyperglycemia was confirmed 24 h prior to implantation surgery with a fasting blood glucose value > 150 mg dL^−1^ (Figure S1, Supporting Information).

### 2.5 Actuation‐Mediated Controlled Release of VEGF Using the SRDD System Stimulates Neovascularization at Implant Sites in Diabetic Rats

**Figure 4:**
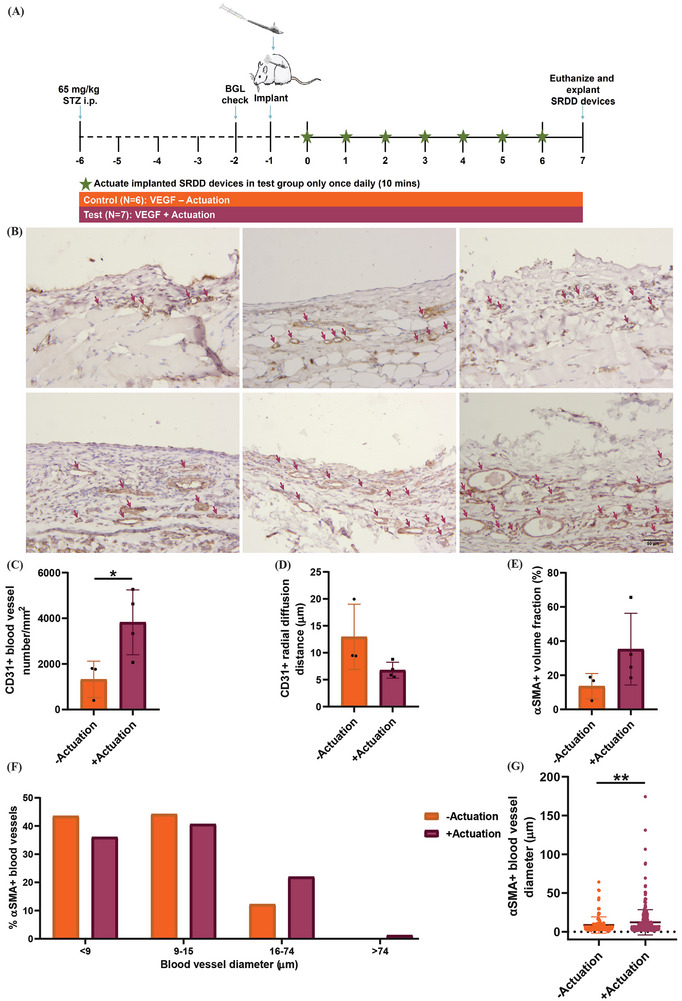
Actuation‐mediated spatiotemporal release of bioactive VEGF stimulates angiogenesis in diabetic rats. A) One SRDD device containing VEGF‐AA‐CMC hydrogel was subcutaneously implanted in 13 rats with implanted devices actuated once daily on day 0, 1, 2, 3, 4, 5, 6, and 7 in the test group. B) Representative images of αSMA staining of vasculature (arrows) surrounding ‐Actuation (top panel) and +Actuation devices (bottom panel), scale bar = 50 µm. C) CD31+ blood vessel number per mm^2^, D) CD31+ radial diffusion distances, E) percentage of total blood vessels expressing αSMA for analysis of vessel stability and maturity. ‐Actuation, *N* = 3; +Actuation, *N* = 4 with data represented as means ± standard deviation, unpaired t‐test with Welch's correction, *p < 0.05. F) α‐SMA+ stained blood vessels. *N* > 200 blood vessels/group with data represented as means only. G) αSMA+ blood vessel diameters, median blood vessel diameter ‐Actuation 5.49 µm and +Actuation 7.12 µm. *N* > 200 blood vessels/group with data represented as means ± standard deviation, unpaired t‐test with Welch's correction, *p < 0.05. AA, alginic acid; CMC, carboxymethylcellulose; BGL, blood glucose level; SRDD, soft robotic drug delivery; STZ, streptozotocin; VEGF, vascular endothelial growth factor.

To investigate if actuation of the SRDD device could spatiotemporally release sequestered VEGF in its bioactive form from the AA‐CMC formulation 2 hydrogel to stimulate angiogenesis locally at implant sites, we implanted SRDD devices loaded with 13.5 µg mL^−1^ VEGF (675 ng VEGF/SRDD device) subcutaneously in the dorsal region of diabetic Sprague‐Dawley rats as depicted in **Figure** **4**A. In one experimental group, the implanted SRDD devices were pneumatically actuated in non‐anaesthetized rats for 7 days with the optimized actuation regime (input pressure of 10 psi for 10 s intervals for a total of 10 cycles) for 10 min every 24 h, using a custom‐made electropneumatic control system to actively release VEGF in a spatiotemporal manner. The other experimental group were the non‐actuated, static controls which depended on passive release of VEGF from the SRDD device containing the hydrogel loaded with VEGF. Immunohistological staining for CD31 and α‐SMA as shown in Figure 4B facilitated the visualization of neovessels and more mature blood vessels, respectively.^[^ ^59^, ^60^ ^]^ A stereological counting technique was applied to the acquired images of the tissue‐device interface (Figure S2A, Supporting Information) for an unbiased estimate of numerical density (Figure S2B, Supporting Information) and radial diffusion distances (Figure S2C, Supporting Information).^[^ ^28^, ^60^, ^61^, ^62^ ^]^ The diameter of each blood vessel counted was also measured (Figure S2D, Supporting Information). The number of α‐SMA+ blood vessels was expressed as a percentage of total blood vessels to determine if a greater proportion of mature blood vessels developed in the presence of actuation‐mediated spatiotemporal release of VEGF compared to passive release of VEGF. Normal distribution was observed in all groups using a Shapiro‐Wilk test and an unpaired t‐test indicated a significant increase in CD31+ blood vessel number per unit area (*p* = 0.0335, Figure 4C) in the actuated group compared to the non‐actuated group. There was no reduction in radial diffusion distances (*p* = 0.2155, Figure 4D) at implant sites in the actuated group compared to the non‐actuated group. There was also no significant difference in the ratio of alpha smooth muscle actin+ (α‐SMA+) to total cluster of differentiation 31+ (CD31+) blood vessels between groups (*p* = 0.0843, Figure 4E). A percentage frequency distribution of mature α‐SMA+ blood vessel diameters showed majority of blood vessels in the actuated group had diameters between 9–15 µm, indicative of small arterioles which branch into capillaries to facilitate exchange of gases, nutrients, and waste products between the bloodstream and surrounding tissue.^[^ ^63^, ^64^ ^]^ The actuation‐mediated spatiotemporal delivery of VEGF produced a higher proportion of mature blood vessels 16–74 µm in diameter compared to the passive VEGF delivery controls (21.99 vs 12.24% α‐SMA+ blood vessels, Figure 4F). A large variation was observed in the measured lumen diameters of CD31+ blood vessels resulting in a non‐normally distributed population by a Shapiro‐Wilk test. The measured lumen diameters of mature α‐SMA+ blood vessels were normally distributed by a Shapiro‐Wilk test and an unpaired t‐test found actuation‐mediated spatiotemporal release of VEGF significantly increased their diameter compared to VEGF passively delivered in the non‐actuated group (*p* = 0.0025, Figure 4G). Thus, daily actuation of the SRDD device released bioactive VEGF in a spatiotemporal manner from the mechanoresponsive hydrogel significantly increasing vessel number and diameter compared to passive delivery of VEGF to stimulate angiogenesis at implant sites in hyperglycemic rats.

### 2.6 Absence of Off‐Target Effects

Systemic administration or uncontrolled dosing of VEGF can result in adverse effects at distant, non‐target locations.^[^ ^40^, ^41^, ^42^, ^65^ ^]^ The SRDD system implanted in this study delivered predetermined amounts of VEGF in a spatiotemporal manner. Hematoxylin and Eosin staining of the additional tissues explanted at euthanasia exhibited no adverse effects from VEGF administration at distal sites, e.g., oedema, hemangiomas (Figure S3, Supporting Information). No difference is observed between the tissues of animals that received VEGF and those that did not, suggesting that localized, controlled delivery of VEGF may help to avoid some of its well‐known off‐target effects.

## 3 Discussion

Presented in this study is the implantable soft robotic drug delivery, SRDD, device that can spatiotemporally control the release of a sequestered therapeutic protein, VEGF, from the mechanoresponsive AA‐CMC hydrogel through the mechanical forces generated during pneumatic actuations. The amount and release pattern of positively charged VEGF from the SRDD system can be controlled by modifying the actuation parameters – compression pressure, ramp, cycle number and profile type (square, trapezoidal, or triangular) as shown in in vitro release studies. We also demonstrate that the SRDD system, used for spatiotemporal delivery of VEGF, can achieve localized angiogenesis at a device implantation site.

Conventional hydrogels exhibit limited mechanical strength and so are unsuitable for repeated drug delivery in response to applied mechanical forces.^[^ ^66^, ^67^, ^68^, ^69^, ^70^ ^]^ Tough hydrogels have increased strength and toughness compared to conventional hydrogels and are developed through polymer reinforcement, interpenetrating polymer networks, covalent and/or non‐covalent cross‐linking.^[^ ^47^, ^71^, ^72^ ^]^ Tough gel, composed of ionically cross‐linked alginate and covalently cross‐linked polyacrylamide, was developed to withstand a high degree of mechanical strain and repeated deformations before material failure.^[^ ^47^, ^71^, ^72^ ^]^ Mendz et al. incorporated this tough gel in their hybrid hydrogel actuator and demonstrated its ability to release a charged model compound, Dextran, in a temporal manner and improve tissue penetration in response to actuation.^[^ ^36^ ^]^ Similarly, we developed a mechanoresponsive AA‐CMC hydrogel that utilizes covalent interactions via adipic acid dihydrazide to enhance mechanical strength. Our negatively charged AA‐CMC hydrogel also incorporates electrostatic interactions via calcium chloride so that amount and rate of positively charged Dextran release could be controlled by modifying the actuation parameters (pressure, ramp, and cycle number) of our SRDD device. Tunable release rates and patterns are particularly important for therapeutic proteins such as VEGF that have a short half‐life in vivo. VEGF requires spatiotemporal delivery to stimulate therapeutic angiogenesis.^[^ ^39^, ^40^, ^42^, ^65^, ^73^, ^74^, ^75^, ^76^ ^]^ We electrostatically interacted VEGF with our mechanoresponsive hydrogel and loaded it into the therapeutic reservoir of the SRDD device with the aim of releasing 5 ng VEGF per day^[^ ^77^ ^]^ to demonstrate that actuation‐mediated release of bioactive VEGF can be achieved in a spatiotemporal manner. It is difficult to recapitulate angiogenesis at an in vitro level due to the complex signaling environment between growth factors and endothelial cells, and many other cells involved in the process of angiogenesis.^[^ ^78^, ^79^ ^]^ For this reason, we focused on the in vivo angiogenic outcomes in this study. We first completed a 7‐day study in healthy rats and found VEGF released from the actuated SRDD system significantly increased CD31+ blood vessel number, length density, and diameter and significantly reduced radial diffusion distance compared to control SRDD systems that were actuated but did not contain VEGF (Figure S4, Supporting Information). Research by Foster et al. is also in agreement as they significantly increased the presence of blood vessels (≈ 60 blood vessels/field of view) in the dorsal subcutaneous space of C57BL/6J mice following the injection of VEGF encapsulated GCRDVPMSMRGGDRCG (VPM)‐cross‐linked microgels. However, VEGF encapsulated dithiothreitol‐cross‐linked microgels were not fully degraded over the 14 days and so did not release their encapsulated VEGF and subsequently had no effect on vascularization. In addition, a bolus of soluble VEGF co‐delivered with empty VPM‐cross‐linked microgels did not increase vascularization.^[^ ^80^ ^]^ Similarly, the SRDD devices in the control group (no VEGF) were also actuated to investigate if device actuation contributed to blood vessel formation. Establishing that device actuation alone, without VEGF, did not significantly increase angiogenesis, allowed comparison of VEGF release from actuated and non‐actuated devices in our diabetic rat study.^[^ ^55^ ^]^

A 7‐day STZ‐induced diabetic rat study was subsequently completed. It is well established that it is difficult to stimulate angiogenesis in diabetes, making this a challenging, and clinically relevant, test for our system.^[^ ^55^, ^56^, ^57^, ^58^ ^]^ The actuation‐meditated release of VEGF from the SRDD system in the hyperglycemic rats resulted in a significant increase in CD31+ blood vessel number and length density at implant sites compared to the passive release VEGF controls. Actuation‐mediated spatiotemporal delivery of VEGF also reduced, although not significantly, radial diffusion distance. Radial diffusion distances are proportional to diffusion times of nutrients, oxygen, and other compounds from an implanted device into the bloodstream. In the context of implanted devices for cell therapies, such as the use of islets in type 1 diabetes mellitus, diffusion time of glucose from the bloodstream to the islets, and of insulin from the islets to the bloodstream, is of critical importance. Therefore, reducing the radial diffusion distance here could have significant positive implications for implantable cell therapy devices.

Interestingly, the actuation‐mediated release of VEGF significantly increased the diameters of α‐SMA+ blood vessels compared to passive release (**p = 0.0023) suggesting that the spatiotemporal release of VEGF can stimulate maturation and further growth of newly formed blood vessels. The majority of the α‐SMA+ blood vessels formed in response to actuation‐mediated release of VEGF had diameters of 9 – 15 µm, which correlates with previous in vitro and in vivo studies that suggest spatiotemporally delivered VEGF also influences lumen diameter.^[^ ^81^, ^82^, ^83^, ^84^ ^]^ Nakatsu et al. detailed that angiogenesis is dose dependent with VEGF concentrations up to 35 ng mL^−1^ stimulating the growth of long, thin blood vessels.^[^ ^85^ ^]^ Higher concentrations of VEGF, similar to what was spatiotemporally delivered in this study, promote the growth of blood vessels with enhanced diameters^[^ ^60^, ^86^ ^]^ as seen with the actuation group producing a higher proportion of α‐SMA+ blood vessels > 15 µm in diameter compared to the passive release of VEGF controls. Golub et al. similarly found that blood vessel diameters were > 10 µm in diameter in female mice administered 8 mg of VEGF‐encapsulated nanoparticles (total VEGF dose 375 ng, compared to 675 ng in our study).^[^ ^87^ ^]^ These findings further demonstrate that our SRDD system has successfully achieved spatiotemporal delivery of VEGF. Our approach demonstrates that soft robotics and their tunable actuation parameters can be used to spatiotemporally release a cationic therapeutic protein, VEGF, to elicit a specific biological response – stimulation of therapeutic angiogenesis. While mechanical stimulation of hydrogels has been trialed before for VEGF release, e.g., by Mooney et al. who compressed calcium cross‐linked alginate hydrogels for local release of bioactive VEGF,^[^ ^9^ ^]^ to‐date these approaches have had little potential for clinical translation. The system presented by Mooney and colleagues was limited by penetration depth of the externally applied mechanical force which would hinder clinical translation. Our SRDD system utilizes soft pneumatic actuators which can be implanted via a minimally invasive procedure to overcome penetration depth of an actuation stimulus for clinical translation. Future work will focus on minimizing the parts of the actuation system so that it can be portable to facilitate progression of the SRDD system to the clinic.

**Figure 5:**
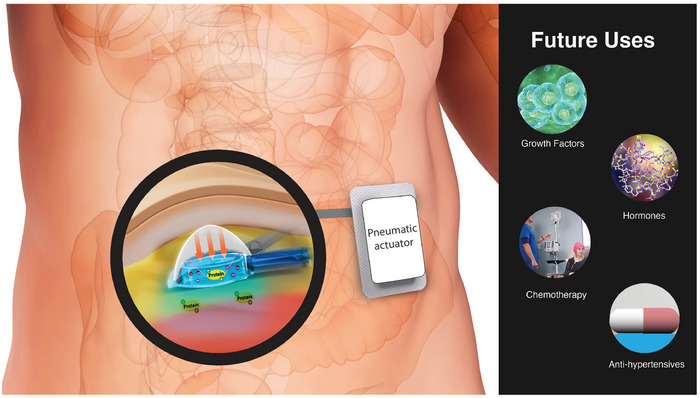
The SRDD system is designed to be implanted subcutaneously and will be connected to an externally worn minimized pneumatic actuator. The system will be used to release a variety of therapeutics which require spatiotemporal delivery to achieve therapeutic efficacy.

In addition to VEGF, several different growth factors and cytokines are involved in therapeutic angiogenesis such as platelet‐derived growth factor, fibroblast growth factor, and hepatocyte growth factor.^[^ ^77^ ^]^ In the future, our SRDD system could be used to deliver a combination of these therapeutic proteins to recapitulate the angiogenic process in vivo. Actuation‐mediated release, while advantageous for therapeutic proteins that require accurate dosing and local controlled delivery, is also beneficial for therapeutic proteins which require a rapid onset, for example rapid‐acting insulin. Despite not being investigated here, the actuation parameters used with the SRDD device in the future could be modified in terms of pressure, ramp, and cycle number to trigger on‐demand and controlled insulin release to maintain blood glucose levels in the narrow normoglycemic range to prevent long‐term complications of hypo‐ or hyperglycemia.^[^ ^88^, ^89^ ^]^ Hence, the SRDD system has the potential to be designed to treat an array of clinical conditions which require spatiotemporal drug delivery (**Figure** **5**).

## 4 Conclusion

In conclusion, this work describes the merging of the nascent field of soft robotics with the well‐established fields of biomaterials and drug delivery. To our knowledge, this is the first report of a fully implantable device, that has demonstrated controlled delivery of a therapeutic protein in vivo using soft robotics resulting in a physiological effect. The SRDD system is more advantageous than current drug delivery systems as it allows for 1) minimally invasive implantation procedure, 2) externally stimulated release of therapeutic proteins, 3) tunable release rates and patterns of therapeutic proteins, 4) potential for non‐invasive therapy refills to extend the therapeutic lifespan of the SRDD device, and 5) potential to deliver a variety of therapeutics.

## 5 Experimental Section

### Materials

All chemicals, reagents, and solvents were purchased as reagent grade from Sigma‐Aldrich or Merck and used as received unless otherwise specified.

### Polymer Synthesis

AA (5.4 g) and CMC (5.24 g) were separately dissolved in 100 mL deionized water by stirring overnight. Once the polymers had completely dissolved, sodium periodate was added at 37 °C to the 25 m solution of AA and 20 m solution of CMC in a molar ratio of sodium periodate: AA/CMC = 1.5:1. The pH of the reaction solution was adjusted to 3 with 1 mol L^−1^ sulfuric acid and stirred at 37 °C for 24 h in the dark. After 24 h, an equimolar amount of ethylene glycol was added and stirred for 1 h to stop the oxidation. The resulting solution was precipitated by adding excess ethanol and centrifuged at 5000 RPM for 10 min at room temperature to collect the pellet. The supernatant was poured away, and the solid pellet was stored in 50 mL Falcon tubes. The pellet was dissolved with the addition of deionized water and the polymers were purified through dialysis (molecular weight cut‐off, 6–8 kDa) in deionized water for 7 days. On day 7, the dialyzed solution was frozen overnight at −80 °C, then freeze dried at −70 °C at 0.000435 psi for 10 days until white amorphous versions of DAA and DCMC were obtained.

### AA‐CMC Based Hydrogel Preparation

The polymers, DAA (1 g) and DCMC (1 g), were separately dissolved in 10 mL deionized water overnight to ensure complete wetting of the lyophilized powders. The cross‐linkers, adipic acid dihydrazide (0.5 m) and calcium chloride (0.2 m), were separately dissolved in deionized water, 40 kDa FITC‐DEAE Dextran, or VEGF (Explora‐Bioscience Srl, Italy). The polymer dispersions, DCMC and DAA, were mixed and added to a glass vial. The cross‐linker dispersions, adipic acid dihydrazide and calcium chloride, with or without FITC‐DEAE‐Dextran or VEGF were also mixed and added to another glass vial. Dispersions were drawn up into individual 1 mL syringes (facilitated keeping cross‐linkers separate until gelation was required) and injected through a y‐mixer (homogenous mixing of polymers and cross‐linkers) into molds or the therapeutic reservoir of the SRDD device (Figure S5, Supporting Information) where gelation would occur within 60–120 s at room temperature.

### Fourier‐Transform Infrared Spectroscopy

Fourier Transform Infrared Spectroscopy was performed on AA, CMC, DAA, DCMC, and cross‐linked AA‐CMC to detect the presence of aldehyde groups.^[^ ^90^, ^91^, ^92^ ^]^ For each sample, 5 mg of lyophilized powder was loaded onto the diamond cell of the spectrometer (Spectrum 400 FTIR spectrometer, Perkin Elmer) and spectra were collected in transmission mode at room temperature over a wavenumber range of 4000–500 cm^−1^ and resolution of 4 cm^−1^.

### X‐Ray Diffraction

X‐ray diffraction was completed on AA, CMC, DAA, and DCMC were loaded into the sample holder of an X‐ray diffractometer (Inel Equinox 6000 Powder X‐Ray Diffractometer) and crystallinity was determined by copper K‐α radiation (1.5406 Å wavelength) in a 2θ range of 10–80°.

### Nuclear Magnetic Resonance

Nuclear magnetic resonance was performed on AA, CMC, DAA, and DCMC. The samples, 20 mg of each, were added to separate 2 mL microcentrifuge tubes. Then 1 mL of deuterium oxide was added to each tube and vortexed until lyophilized forms were fully dissolved. A further 1 mL of deuterium oxide was added to all samples which were then left overnight at 4 °C on the tube roller. The samples were then added to clean, dry tubes and placed in the sample chamber of the spectrometer (Agilent DD2 NMR 600 MHz 54 mm ASC Spectrometer). Proton nuclear magnetic resonance analysis was completed on all samples with measurements carried out at 19–20 °C.

### Aldehyde Assay

First, 50 mL aqueous solution of 10% w/v DAA/DCMC and 10.1 mL of 0.25 mol L^−1^ sodium periodate was added to a 100 mL glass bottle covered with aluminum foil and left spinning at room temperature. Aliquots of 0.3 mL were removed at predetermined timepoints and diluted to 100 mL using deionized water. Then 0.5 mL of the diluted aliquots was transferred to a cuvette containing 1.0 mL of the indicator solution (1:1 solution of aqueous potassium iodide (20% w/v potassium iodide in sodium phosphate buffer pH 7) and thyodene solution (10% w/v thyodene in sodium phosphate buffer pH 7). The cuvette was then placed in the spectrometer (Varian Cary 100 UV/VIS Spectrometer) and concentration of unreacted periodate measured spectrophotometrically at 486 nm. This value was then subtracted from the original concentration of periodate added to determine the percentage oxidation of DAA and DCMC polymers.

### Zeta Potential

Zeta potential was carried out on AA and CMC, DAA and DCMC, which were prepared at a concentration of 10% w/v in deionized water. Samples were loaded to the cuvettes, placed into the sample holder of the Zetasizer (Zetasizer Nano ZS of Dynamic Light Scattering System), and an electric field was applied to determine surface charge of the polymers AA and CMC and their dialdehyde versions, DAA and DCMC.

### Rheology

Strain sweeps were completed on different formulations (Table 1) of the AA‐CMC hydrogel to determine the linear viscoelastic range of the sample. Strain sweeps were performed on AA‐CMC hydrogel formulation 1, 2, and 4 using the rheometer (MCR302 Rheometer Anton Paar) set to a strain range of 0.01–10% using a 10 mm probe with the parallel plate set at a 2.0–2.2 mm gap and temperature set to 25 °C. Parameters to be keep constant in subsequent frequency tests were selected from the obtained linear viscoelastic range. Frequency sweeps were then performed on the different AA‐CMC hydrogel formulations to determine the time required for gelation to occur. The storage modulus (G’) and loss modulus (G’’) were measured at 0.1% strain and oscillations of 0.1–100 Hz frequency. Hydrogel formation was defined as G’ exceeding G’’, indicating that the elastic properties of the sample were predominant.^[^ ^93^ ^]^

### Mechanical Compression Testing

Three hydrogel discs per formulation (Table 1) were obtained by adding 200 µL of each formulation into individual wells of the in‐house created molds. Hydrogel discs solidified within 60–120 s and were transferred from molds and stored in labeled petri dishes containing 2 mL of 1 X phosphate buffered saline for at least 2 h. The hydrogel discs were transferred to the plate of the mechanical testing machine (Z050 Universal Testing Machine Zwick/Roell) and compressive force of 40% at a rate of 0.01 mm s^−1^ was applied using a 10 N load cell with Young's modulus calculated between 10 and 20% compression. The optimal formulation of the AA‐CMC hydrogel was selected based off the formulation with the highest Young's modulus obtained in the compression tests. This formulation was then subjected to cyclic compression testing. The 200 µL hydrogel disks were placed on the stage of the Zwick and subjected to 40% compression at a frequency of 1 Hz for a total of 10 cycles. The maximal force required to compress the hydrogel on the first cycle was compared to that required on the last cycle to assess damage caused to the hydrogel by the cyclic loading.

### Inverted Tube Test

The AA‐CMC formulation 2 was selected and prepared in a 2 mL glass vial. Gelation was deemed to have occurred when the formulation components remained at the bottom of the vial for 30 s following inversion of the vial. The time taken for gelation to occur was noted to ensure the dispersion would remain viscous for sufficient time to inject through the therapeutic catheter of the SRDD device and then gelate within the therapeutic reservoir.

### SRDD Device Fabrication

Thermoplastic polyurethane (TPU, 0.3 mm and 0.15 mm, MT‐1001‐C and HTM‐8001‐M polyether TPU film, American Polyfilm, Inc.) was vacuum thermoformed over in‐house 3D printed positive molds (RS‐F2‐GPGR‐04, photopolymer resin, Formlabs) (Figure S6, Supporting Information). Five pores with 0.3048 mm diameter were laser cut into a 0.15 mm TPU membrane (Figure S7, Supporting Information) using a carbon dioxide 30 W aluminum laser at 10600 nm wavelength (Trotec Speedy 100). The thermoformed and laser cut TPU membranes were assembled into the 3D printed negative mold with Teflon (MDP Supplies) placed in the catheter channels to prevent heat sealing (Figure S8, Supporting Information). The assembly was heat sealed at 330°F using a heat transfer machine (330QXAi, PowerPress). TPU catheter tubing (0.9 mm inner diameter, MRE037 Micro‐Renathane, Braintree Scientific) of 4.5 cm (therapeutic catheter) and 9 cm (actuation catheter) lengths were inserted over perfluorooctanoic acid‐polytetrafluoroethylene treated mandrels (0.508 mm outer diameter, Tegra Medical) placed in the catheter channels and heat sealed to the device using heat shrink tubing (inner diameter = 1.6 mm, RNF100, RS Ireland). The fully assembled rat scaled SRDD devices measured 3 mm in height and 6 mm in diameter and consisted of a therapeutic and actuation reservoir (Figure S9, Supporting Information). All SRDD devices for preclinical studies were sterilized using ethylene oxide.

### Electropneumatic Actuation and Control System

A custom‐made electropneumatic actuation system was pre‐programmed to utilize electrical signaling to control pneumatic power sources. A positive pressure generator (3‐4 oil‐lubricated piston compressor, Jun‐Air) and vacuum (VP18 Plus, LabTech) were controlled and regulated by an electrically powered controller (OB1 MK3+ microfluidic flow controller, Elveflow) to pneumatically actuate the SRDD devices. The compressor could work at 0–230 psi, the vacuum at 0–8.7 psi, but the controller provided a pressure range channel of ‐14.5–14.5 psi. Both the compressor and vacuum were placed in series with the inflow ports of the controller while the SRDD device was placed in series with the outflow tube of the controller. Actuation of an SRDD device was achieved by loading the actuation reservoir with positive pressure to deflect the therapeutic membrane downwards and then applying a negative pressure to deflate the actuation reservoir and return the therapeutic membrane to its native state. The actuation parameters (pressure (psi), duration of the compression, plateau, and decompression ramps (sec), number of cycles, and “sleeping mode” duration (sec)) were controlled by the ESI Elveflow software interface that drives the controller by uploading.txt format profiles written in MatLab. A manifold (LVF‐KMM‐02, Elveflow) was attached to the outflow tubing of the controller to actuate SRDD devices implanted in separate rats simultaneously, ensuring the set pressure levels were consistently achieved.

### In Vitro Release Studies

Initial in vitro drug release studies to optimize the actuation regime were conducted using a model drug, Fluorescein isothiocyanate–Diethylaminoethyl–Dextran (FITC‐DEAE‐Dextran) of the same charge (cationic) and molecular weight (40 kDa) as Recombinant Human Vascular Endothelial Growth Factor, rHuVEGF165 (VEGF, 202 012 Explora Bioscience). The SRDD devices were weighed before and after loading with the AA‐CMC hydrogel delivering FITC‐DEAE‐Dextran, to facilitate calculation of total hydrogel loaded into SRDD devices and then the percentage of drug subsequently released during experiments. SRDD devices were submerged in microtubes containing 1 mL of release media (1 X phosphate buffered saline, pH 8), connected to the electropneumatic actuation system and actuated once hourly for 5/7 h or once daily for 7 days (to replicate the 7‐day in vivo studies) using customizable actuation regimes. After actuation at each of the predetermined timepoints, SRDD devices were transferred to microtubes containing 1 mL of fresh release media, while microtubes of the released medium were stored at −20 °C. The concentration of FITC‐DEAE‐Dextran in released samples was determined by adding 200 µL of release medium, in triplicate, to a black 96‐well plate and absorbance was read at 528 nm with unknown concentrations interpolated from the standard curve. The actuation parameters were modified until the optimal pressure, ramp, and cycle number were determined (based on pre‐determined optimal VEGF release criteria).

### In Vivo Preclinical Studies

Animal procedures were reviewed and approved according to ethical regulations governed by the Italian Ministry of Health (938/2021‐PR). Animals were housed with a 12 h on/off light cycle, at 20–22 °C and 30–70% relative humidity. Animals were singly housed with standard bedding and food for the duration of the study. Ethylene oxide sterilized SRDD devices were loaded with the AA‐CMC hydrogel with 13.5 µg mL^−1^ VEGF before implantation.

### Diabetic Rat Preclinical Study

Angiogenesis is more difficult to stimulate in diabetes^[^ ^55^, ^56^, ^57^, ^58^ ^]^ and so a diabetic rat model was established to determine whether actuation of the SRDD system produced a superior angiogenic effect compared to diffusion from the SRDD system without actuation. On day ‐6, female Sprague Dawley rats (aged 11 weeks, 250–310 g, Charles Rivers Laboratories, Italy) were weighed and fasted for 2 h before administering 65 mg kg^−1^ STZ (10–2127 Focus biomolecules) intraperitoneally. Rats were returned to their individual cages and provided access to 600 mL of 10% w/v sucrose solution (B000035 Fresenius Kabi) for 48 h to reduce the risk of STZ‐induced hypoglycemia. On day ‐2, all rats were fasted, weighed and blood glucose levels measured to determine onset of diabetes with fasting blood glucose > 150 mg dL^−1^ considered diabetic. On day ‐1, STZ‐induced type 1 diabetic rats were anesthetized using ketamine (70 mg kg^−1^) and medetomidine (0.5 mg kg^−1^) and the SRDD device was subcutaneously implanted in the dorsal region of the rat (Figure S10, Supporting Information). The surgical site was prepared by removing the hair on the back of the rat using a clippers and disinfecting with 70% ethanol. An anterior incision was made at the base of the neck for the transcutaneous port (VAB95BS‐MRI, Instech laboratories) and a posterior incision was made 7 cm from the original incision and 1 cm lateral of the spine for the SRDD device. A blunt dissection was made at both incisions, and the 3 × 6 mm SRDD device was tunneled from the anterior incision subcutaneously into the posterior position using a forceps and its actuation catheter was connected to the transcutaneous port positioned at the base of the neck (Figure S10, Supporting Information). The incision sites were closed with interrupted sutures (3–0 monofilament) and the rat was allowed to recover on a heated pad. The rat was administered 300 µL of warm saline subcutaneously to replete intraoperative fluid losses and then returned to its individual cage with water and food *ad lib* to recover from the implantation procedure.

### Actuation‐Mediated Release of VEGF

At 1 day following implantation, the SRDD actuation reservoir was connected to the custom‐made pneumatic control unit, via the subcutaneous self‐sealing access port, using a VAH6M connecter (Instech) and pneumatically actuated for 10 cycles of 10 psi, 10 s on and 90 s off. The SRDD devices were not actuated in the passive VEGF controls.

### Tissue Processing and Histological Analysis

Blood glucose levels were measured by tail vein sampling at day 7 in the STZ‐induced diabetic rats. Rats were anesthetized with ketamine (70 mg kg^−1^) and medetomidine (0.5 mg kg^−1^) on day 7 and macroscopic examination of the implant site was carried out. An overdose of ketamine and medetomidine was subsequently administered to euthanize rats (normal anesthetic dose is 100 µL 100 mg^−1^ versus euthanasia dose is 200 µL 100 mg^−1^). Explanted SRDD devices and surrounding tissues were fixed for 24 h in 10% formalin (pH 7.4). Fixation, embedding, and staining preparation and imaging were previously described in detail by Dolan et al.^[^ ^28^ ^]^ Fixed tissue samples (*n* = 3 for control group and *n* = 4 for treatment group in diabetic rat study, but *n* = 5 for control group and *n* = 6 for treatment group in healthy rat study (Supporting Information)) were oriented and embedded in paraffin wax blocks for histological analysis. Sections of 5 µm were cut, deparaffinized in xylene, and rehydrated through a series of graded alcohols. For histological analysis of angiogenesis, primary antibodies of CD31 (1:1000, AB182981 Abcam) or α‐SMA (1:1000, A2547 Sigma‐Aldrich) were incubated overnight at 4 °C. Tissue sections were then stained with 3,3′‐Diaminobenzidine solution (GV825 Dako) (EnVision FLEX 3,3′‐Diaminobenzidine Chromogen was mixed with EnVision FLEX Substrate Buffer) and counterstained with Hematoxylin (C0302 Diapath) as per manufacturer instructions. Sections were cover slipped using mounting medium (06 0200 Diapath) and imaged on a Zeiss Microscope combined with Zeiss software.

### Analysis of Angiogenesis

Angiogenesis was assessed as previously described by Dolan et al.^[^ ^28^ ^]^ Briefly, a stereological counting technique was applied to CD31‐ and α‐SMA‐stained images providing unbiased estimates of numerical density (Na) (Figure S2B, Supporting Information), length density (Lv) (Figure S2C, Supporting Information) and radial diffusion distances (Rd) (Figure S2D, Supporting Information).^[^ ^62^ ^]^ To further assess the angiogenic response to controlled delivery of VEGF, the number of blood vessels stained positive for α‐SMA were expressed as a percentage of total blood vessels counted. Blood vessels were also measured for vessel diameter analysis (≥ 200 vessels per animal).

### Statistical Analysis

GraphPad Prism (8.0.1) was used for statistical analysis. Normality was tested using Shapiro‐Wilk test, but nonparametric tests were sometimes performed for *n* ≤ 3 per group, in part because the sample was too small to test whether the endpoint was normally distributed. Unpaired t‐tests were carried out for normally distributed data. A one‐way or two‐way ANOVA with post‐hoc Tukey's multiple comparison was used to compared two groups. A Mann‐Whitney U was performed on not normally distributed data (or a Kruskal‐Wallis test was used with Dunn's post hoc adjustment for multiple comparisons). Statistical significance was accepted when *p* < 0.05.

## Conflict of Interest

The authors declare no conflict of interest.

## Supporting information



Supporting Information

Supplemental Movie 1

Supplemental Movie 2

Supplemental Movie 3

## Data Availability

The data that support the findings of this study are available from the corresponding author upon reasonable request.
