# Supplementary material for: Actuation‐Mediated Compression of a Mechanoresponsive Hydrogel by Soft Robotics to Control Release of Therapeutic Proteins
Source: Adv Sci (Weinh). 2024 Dec 18;12(7):2401744. doi: 10.1002/advs.202401744 (PMC11831469; doi:10.1002/advs.202401744)
Supplement: Supplementary file 1 — Supporting Information [file ADVS-12-2401744-s003.docx]

Supporting Information

Title Actuation-mediated compression of a mechanoresponsive hydrogel by soft robotics to control release of therapeutic proteins

Eimear J. Wallace*, Joanne O’Dwyer, Eimear B. Dolan, L. P. Burke, Robert Wylie, Gabriella Bellavia, Stefania Straino, Francesca Cianfarani, Gabriella Ciotti, Simona Serini, Gabriella Calviello, Ellen T. Roche, Tapas Mitra*, and Garry P. Duffy*

**Supplementary materials and methods**

Refilling the therapeutic reservoir of the SRDD device: The SRDD devices were weighed before and after loading with the AA-CMC hydrogel delivering methylene blue (M9140 Merck), hydrogel 1, and AA-CMC hydrogel delivering Fluorescein isothiocyanate–Diethylaminoethyl–Dextran (FITC-DEAE-Dextran), hydrogel 2, to facilitate calculation of total hydrogel loaded into SRDD devices and then the percentage of drug subsequently released during experiments. The SRDD device was submerged in a beaker of DI H_2_O, connected to the electropneumatic actuation system, and actuated once hourly for 7 h using the customizable actuation regime (70 consecutive *in vitro* actuations). After actuation the SRDD device was weighed and then refilled with hydrogel 2 to demonstrate the potential to refill the SRDD device.

Non-diabetic rat preclinical study: Animal procedures were reviewed and approved according to ethical regulations governed by the Italian Ministry of Health (719/2020-PR). Animals were housed with a 12 h on/off light cycle, at 20-22°C and 30-70% relative humidity. Animals were singly housed with standard bedding and food for the duration of the study. Ethylene oxide sterilized SRDD devices were loaded with the AA-CMC hydrogel with 13.5 µg/mL or without VEGF before implantation. To determine the bioactivity of VEGF released in a spatiotemporal manner from the SRDD system female Sprague Dawley rats (350-450 g, Charles Rivers Laboratories, Italy) were anaesthetized using ketamine (70 mg/kg) and medetomidine (0.5 mg/kg) and the SRDD device was subcutaneously implanted in the dorsal region of the rat as described above. The rat was administered 300 µL of warm saline subcutaneously to replete intraoperative fluid losses and then returned to its individual cage with water and food *ad lib* to recover from the implantation procedure.

Actuation-mediated controlled release of VEGF using the SRDD system stimulates neovascularization at implant sites in non-diabetic rats: To investigate if actuation of the SRDD device could release VEGF in its bioactive form to stimulate angiogenesis locally at implant sites, we implanted SRDD devices loaded with AA-CMC hydrogel with or without 13.5 µg/mL VEGF subcutaneously in the dorsal region of non-diabetic Sprague-Dawley rats as depicted in **Figure S4A**. The implanted SRDD devices were pneumatically actuated in non-anaesthetized rats for 7 days with the optimized actuation regime (10 cycles of 10 psi input pressure, 10 sec on and 90 sec for 10 min every 24 h) using a custom-made electropneumatic control system. Immunohistological staining with CD31 and α-SMA as shown in **Figure S4B** facilitated the visualization of neovessels and more mature blood vessels, respectively^[59,60]^ with the aforementioned stereological counting technique employed to assess the vascularity at the tissue-SRDD device porous membrane interface. Normal distribution was observed in all groups using a Shapiro-Wilk test and an unpaired t-test was performed on histological data. Histological assessment at the tissue-SRDD device porous membrane interface indicated a significant increase in CD31+ blood vessel number per unit area (p = 0.0310, **Figure S4C**) and decrease in radial diffusion distances (p = 0.0460, **Figure S4D**) in the presence of VEGF compared to the absence of VEGF. There was no significant difference in the ratio of α-SMA+ to total CD31+ blood vessels between groups (p = 0.3030, **Figure S4E**). A percentage frequency distribution of blood vessel diameters shows that the majority of blood vessels had diameters between 9-15 μm with a greater proportion of blood vessels grown in the presence of VEGF in that category compared to no VEGF (45.98 vs 39.53% CD31+ blood vessels, **Figure S4F**). A non-parametric t-test performed with a subsequent Mann-Whitney U analysis found the diameters of α-SMA+ blood vessels formed in the presence of VEGF were not significantly greater (p = 0.4720) than those formed in the absence of VEGF. However, the diameters of CD31+ blood vessels formed in response to VEGF were significantly greater (p = 0.0130, **Figure S4G**) than those formed in the absence of VEGF. This indicated an increased vascularity of the tissue underlying the porous membrane of the SRDD device after actuation-mediated controlled release of bioactive VEGF.

**Supplementary figures**

**
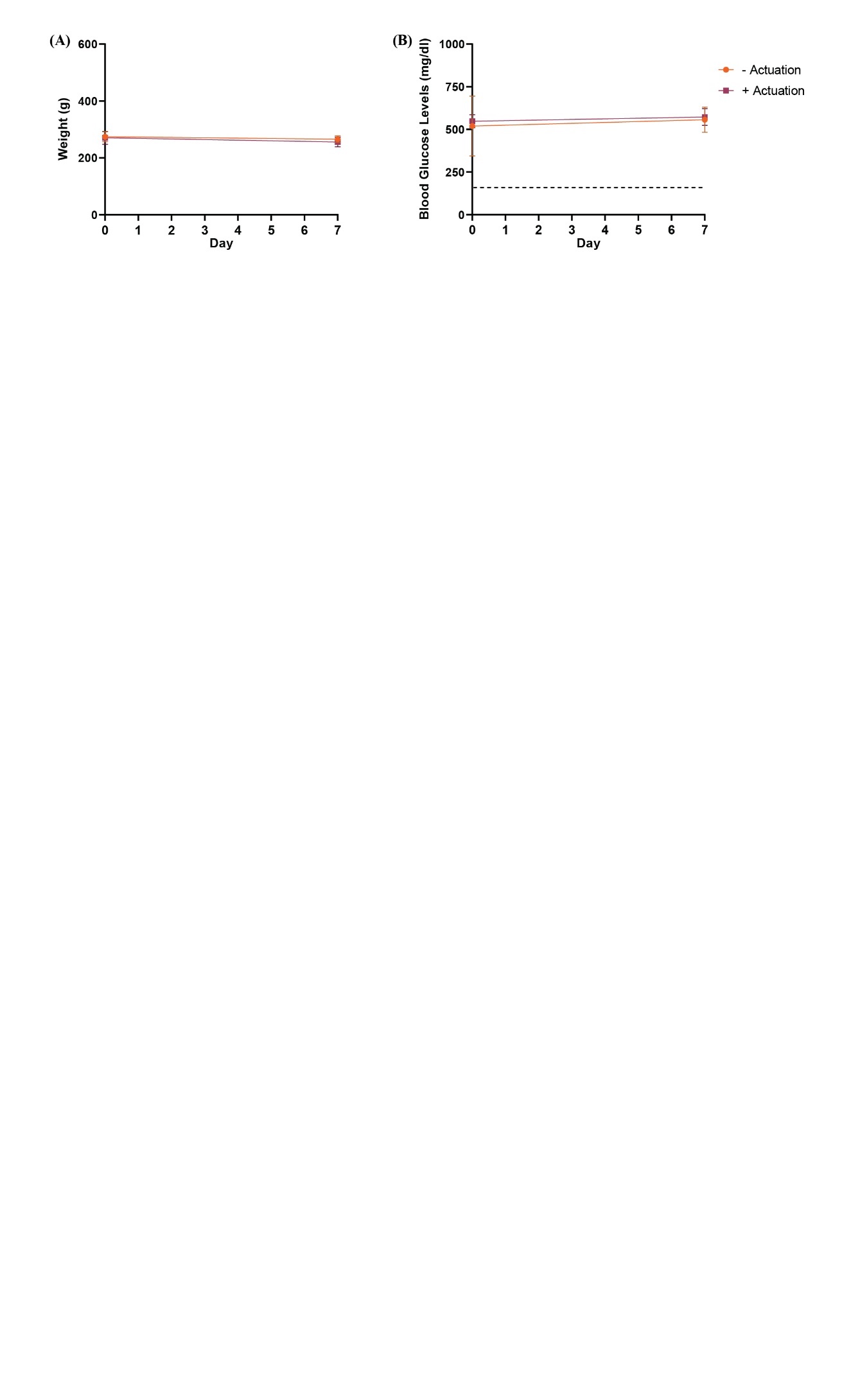
**

Figure S1 (A) Body weight of rat before SRDD device implantation (day 0) until euthanasia (day 7). Mean ± standard deviation, unpaired t-test with Welch’s correction. (B) Blood glucose levels of rats before SRDD device implantation (day 0) until euthanasia (day 7) with fasting blood glucose > 150 mg/dL considered diabetic (cut-off represented by dashed line). Mean ± standard deviation, unpaired t-test with Welch’s correction. -Actuation, N = 6; +Actuation, N = 7.


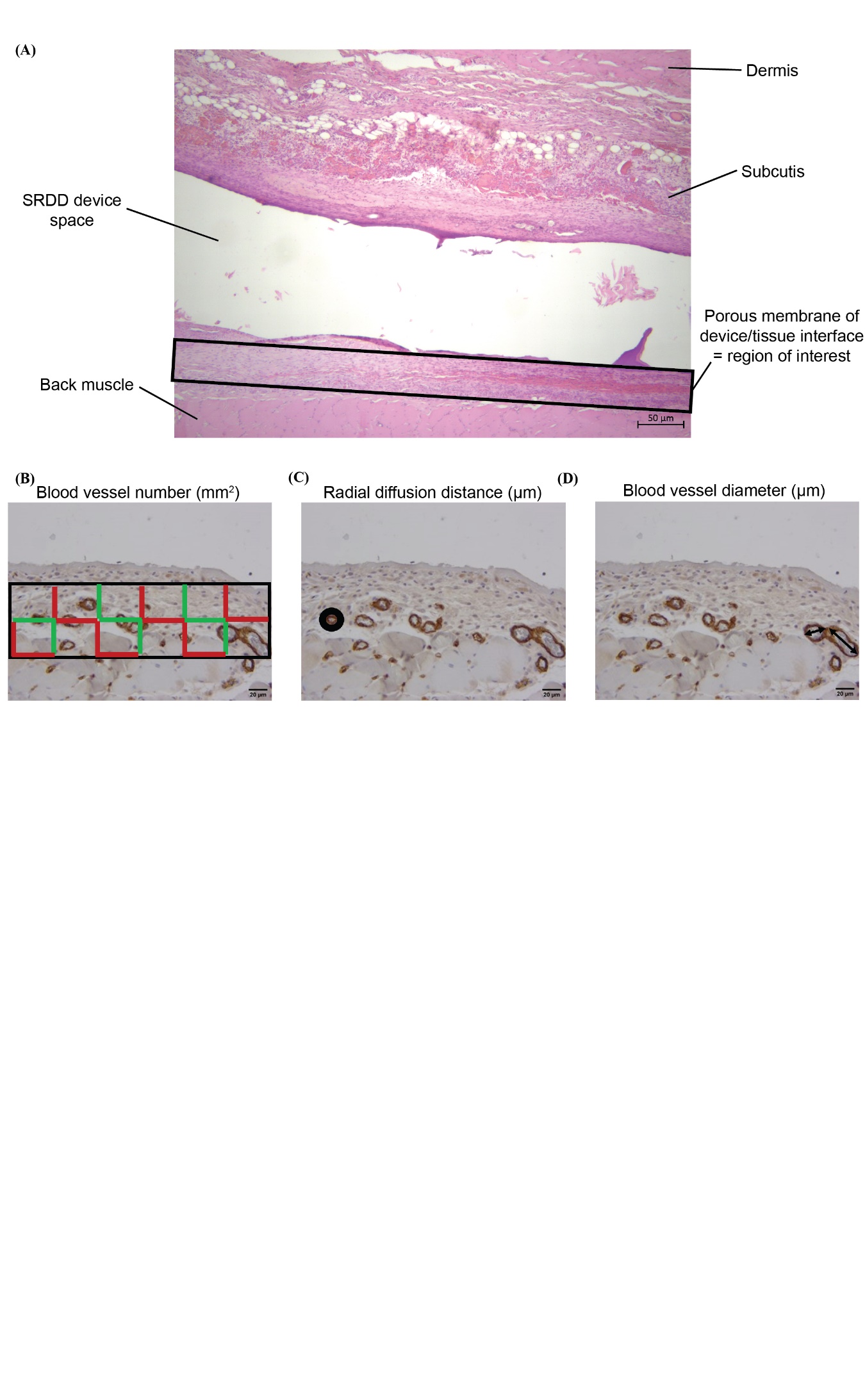


Figure S2 (A) Hematoxylin and Eosin-stained tissue-device interface region of interest for angiogenesis analysis. 4X magnification. Scale bar = 50 μm. (B) Stereological counting frame applied to tissue of interest to calculate blood vessel number, (C) radial diffusion distance, and (D) diameters of blood vessels.


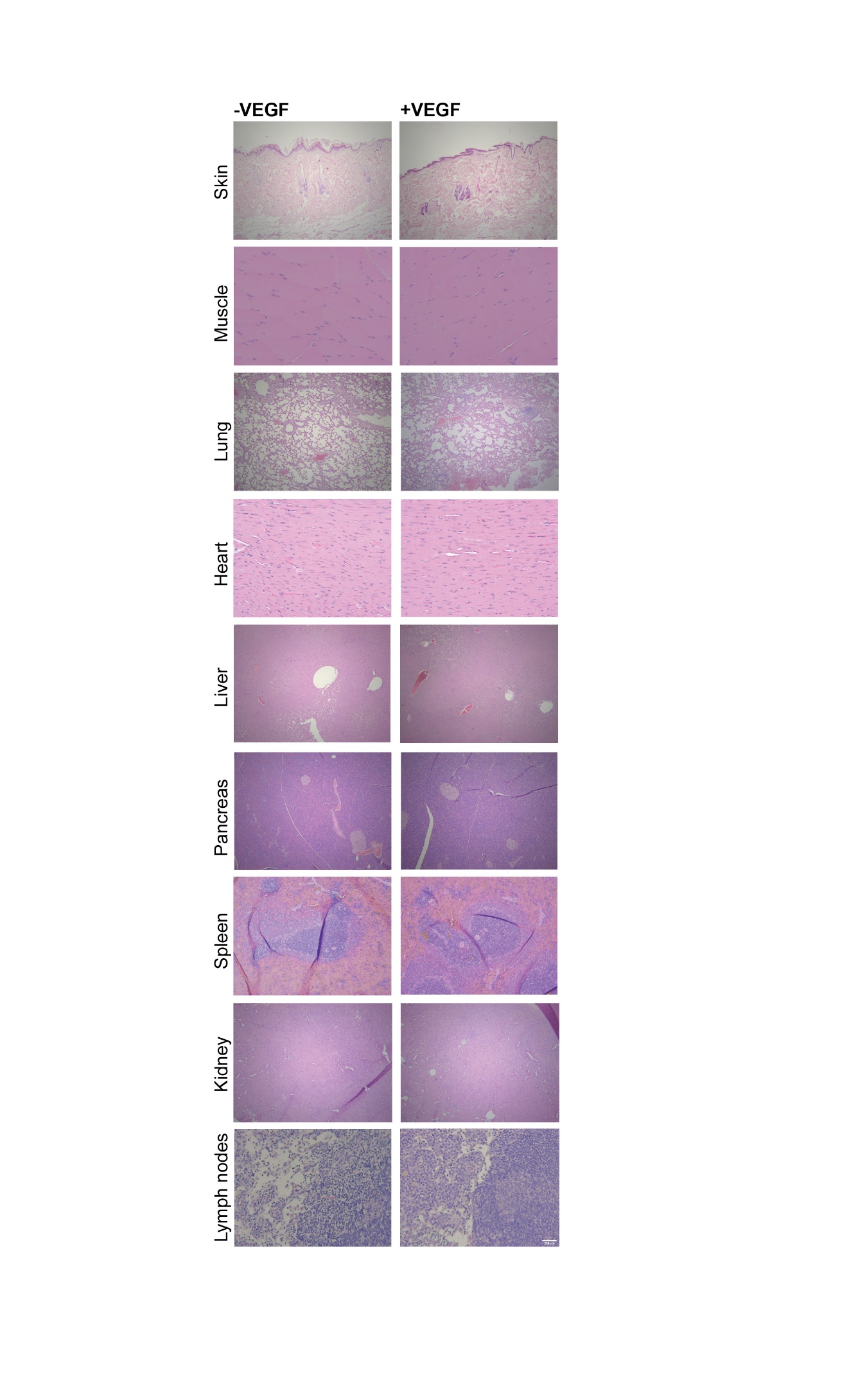


Figure S3 Spatiotemporal release of VEGF does not result in off-target effects in the tissues tested. Panel of Hematoxylin and Eosin-stained tissues at 20 X magnification, Scale bar = 200 µm. VEGF, vascular endothelial growth factor.


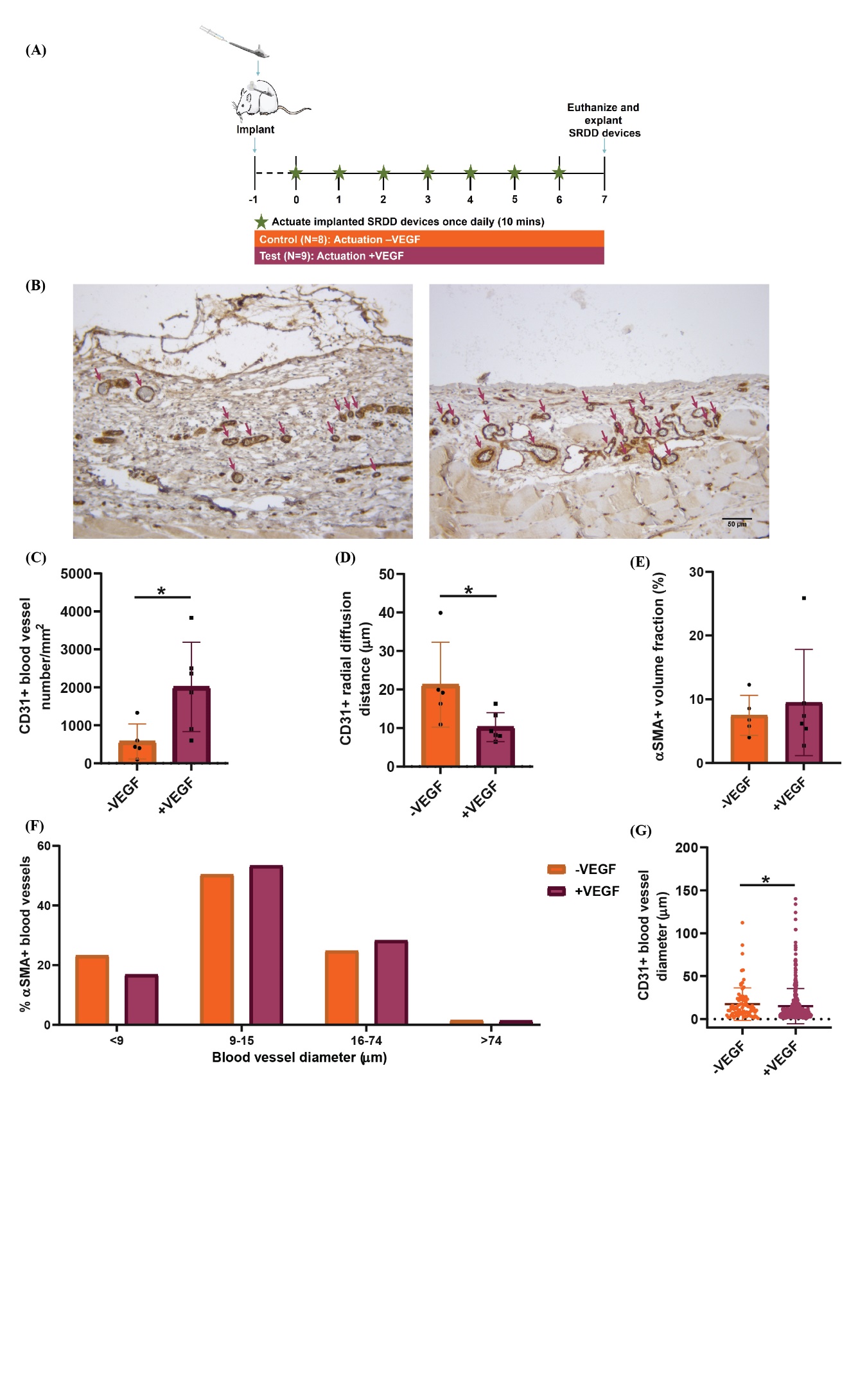


Figure S4 Actuated-mediated release of bioactive VEGF stimulates angiogenesis in non-diabetic rats. (A) Preclinical non-diabetic rat study overview. (B) Representative images of CD31 staining of vasculature (arrows) surrounding -VEGF (left) and +VEGF devices (right), scale bar = 50 μm. (C) CD31+ blood vessel number per mm^2^, (D) CD31+ radial diffusion distances, (E) percentage of total blood vessels expressing αSMA for analysis of vessel stability and maturity. -VEGF, N = 5; +VEGF, N = 6 with data represented as means ± standard deviation, unpaired t-test with Welch’s correction, * p < 0.05. (F) α-SMA+ stained blood vessels. N > 200 blood vessels/group with data represented as means only. (G) CD31+ blood vessel diameters, median blood vessel diameter -VEGF 12.07 μm and +VEGF 7.84 μm. N > 200 blood vessels/group with data represented as means ± standard deviation, unpaired t-test with Welch’s correction, * p<0.05. VEGF, vascular endothelial growth factor.


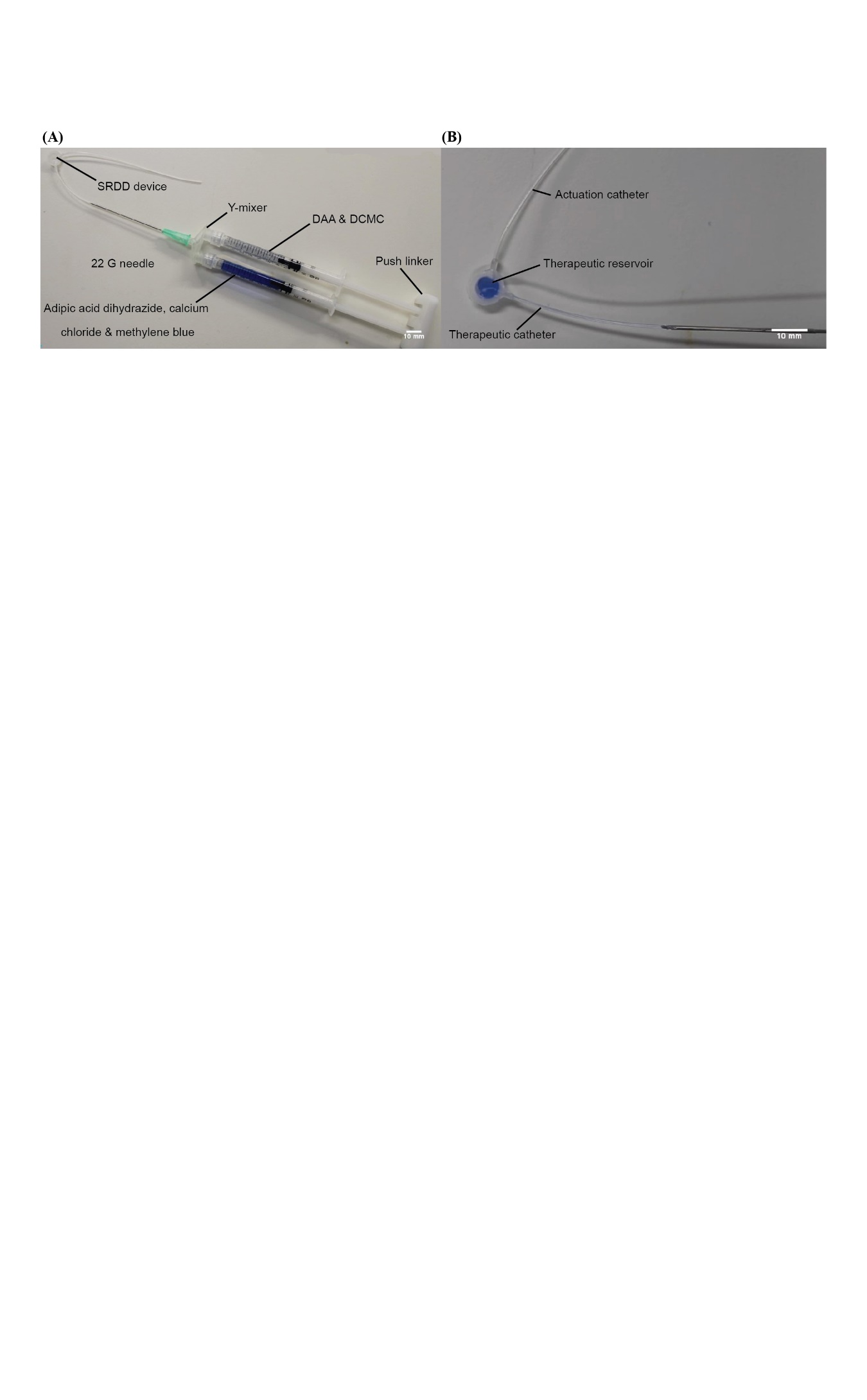


Figure S5 (A) Double barrel syringe set up to expel equal amounts of hydrogel components to form AA-CMC hydrogel for *in vitro* release studies, (B) injecting AA-CMC hydrogel formulation into therapeutic reservoir of SRDD device where it will gel. ADH, adipic hydrazide; CaCl_2_, calcium chloride; DAA, dialdehyde alginic acid; DCMC, dialdehyde carboxymethylcellulose; SRDD device, soft robotic drug delivery device. Scale bar = 10 mm.


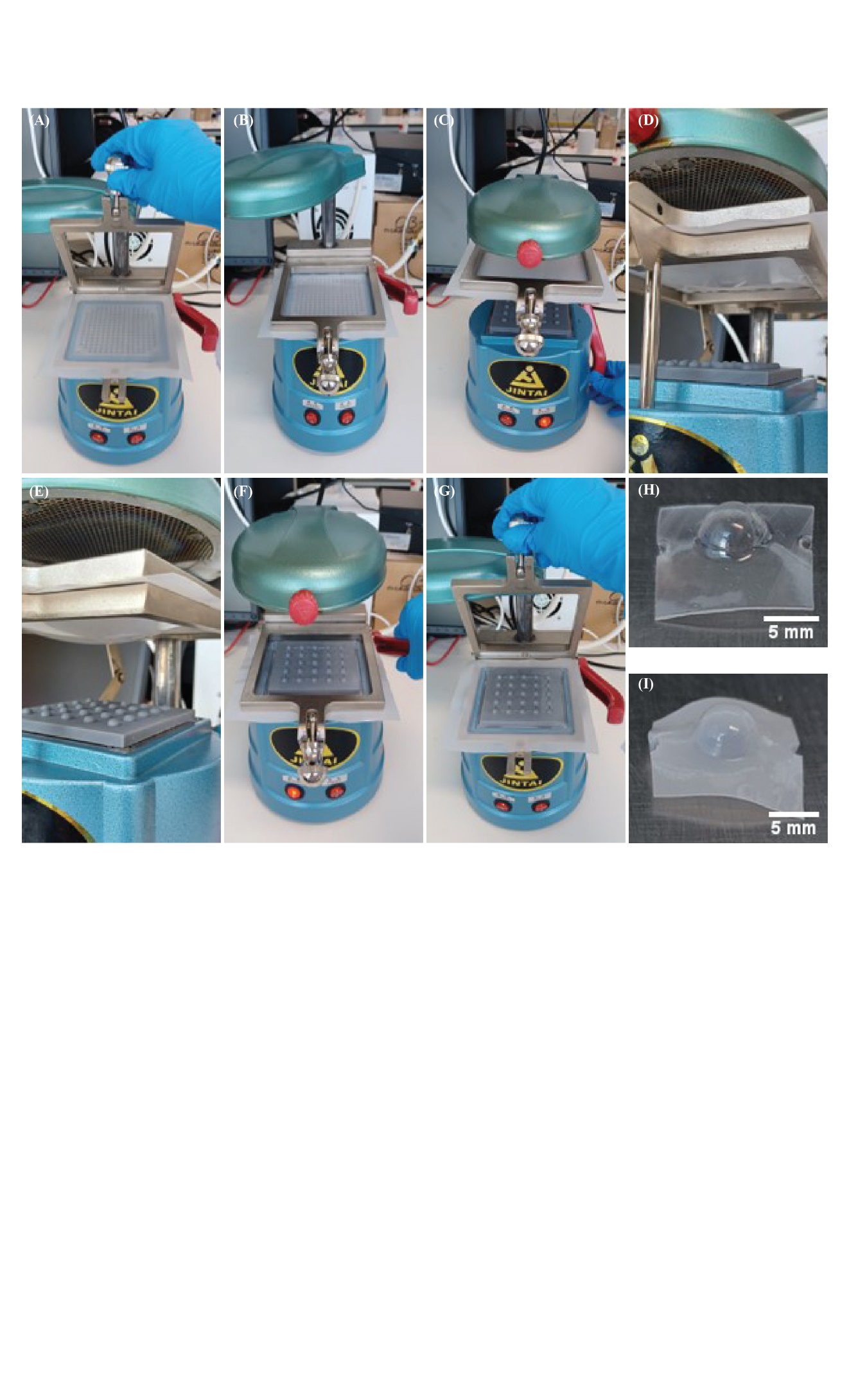


Figure S6 Overview of the procedure for forming TPU reservoirs using a vacuum thermoformer. (A) TPU placed on stage of vacuum thermoformer, (B) TPU sheet clamped into place, (C) TPU sheet raised to heat element of thermoformer and 3D positive mold placed on stage, (D) under view of TPU sheet being heated (note flat appearance), (E) TPU sheet begins to sag, (F) heat turned off, vacuum turned on, and TPU sheet lowered onto positive 3D mold, (G) TPU reservoirs forming on 3D positive mold, (H) 0.30 mm TPU individual reservoirs for actuation reservoir, (I) 0.15 mm TPU individual reservoirs for therapeutic reservoir. Scale bar = 5 mm. TPU, thermoplastic polyurethane.


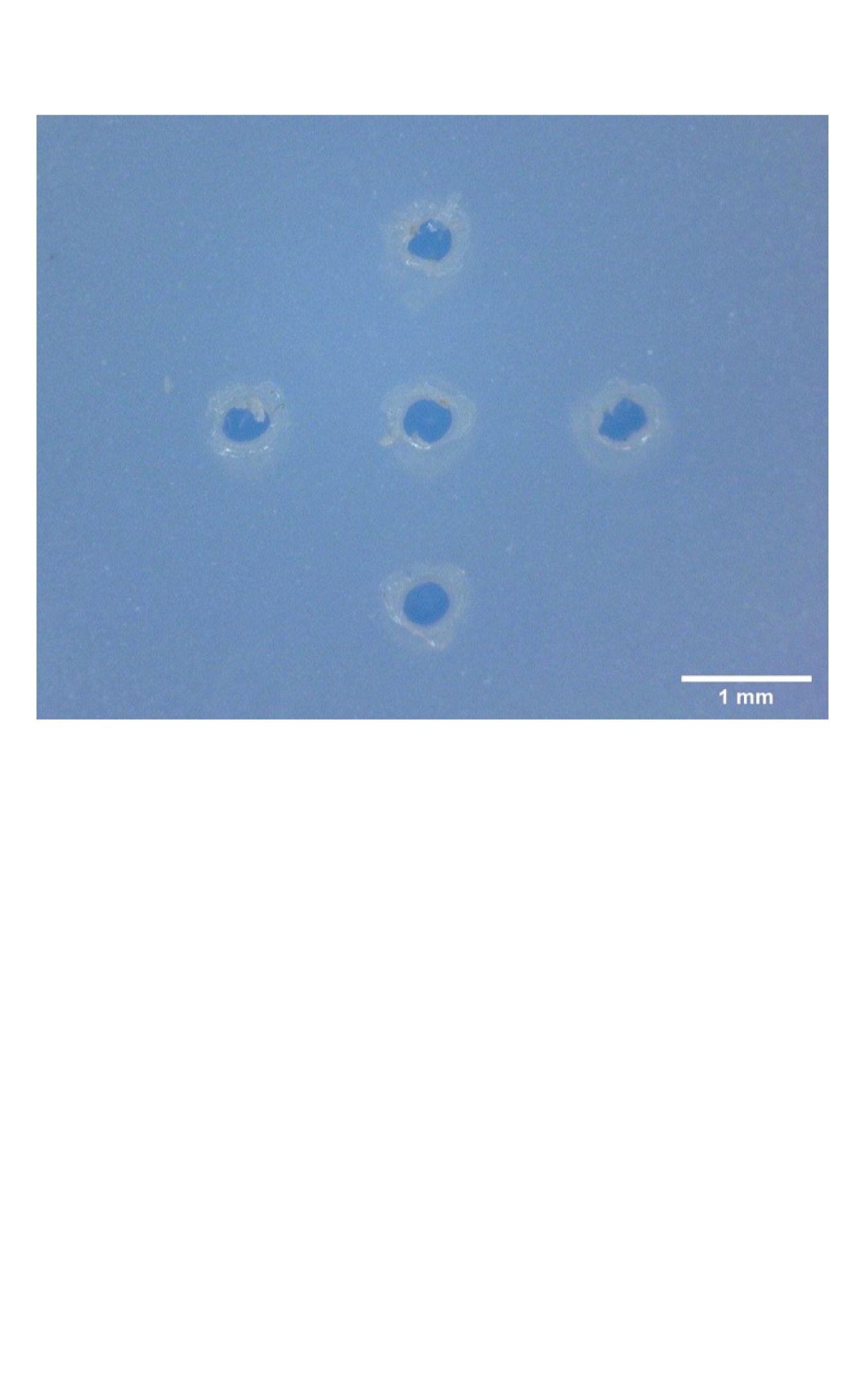


Figure S7 Five evenly distributed 0.3048 mm diameter, non-rate limiting pores in 0.15 mm thermoplastic polyurethane. Scale bar = 1 mm.


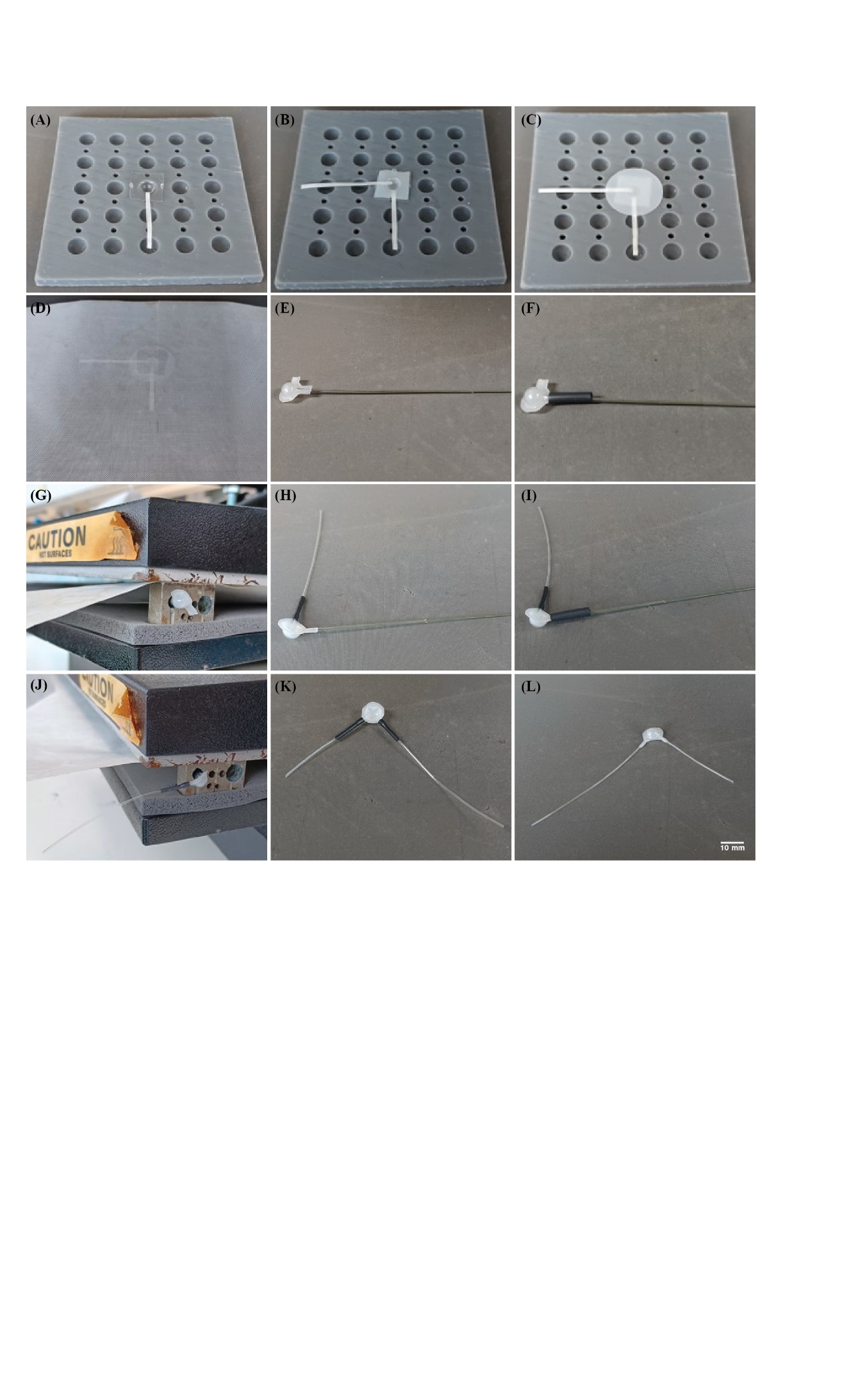


Figure S8 Overview of the fabrication procedure of the SRDD device. (A) 0.3 mm TPU actuating reservoir was placed in the negative mold with a Teflon strip (keeps channel for actuation catheter open during heat sealing), (B) 0.15 mm TPU therapeutic membrane added on top of 0.3 mm actuating reservoir and Teflon strip placed in position of therapeutic catheter, (C) 0.15 mm laser cut TPU with 5 evenly distributed non-rate limiting pores were placed on top of therapeutic reservoir, (D) Teflon sheet placed on top of TPU components and heat pressed at 165˚C for 4 s, (E) 7 cm polyurethane catheter tubing (with perfluorooctanoic acid-polytetrafluoroethylene treated mandrel (0.508 mm outer diameter, Tegra Medical) inside to prevent catheter closing during heat sealing) placed into actuation channel, (F) 1.6 mm heat shrink tubing slipped over SRDD device/catheter junction, (G) actuation catheter placed in aluminum block and heated at 165˚C for 120 s, (h) 4.5 cm polyurethane catheter tubing (with mandrel inside) placed into therapeutic channel, (I) 1.6 mm heat shrink tubing slipped over SRDD device/catheter junction, (J) therapeutic catheter placed in aluminum block and heated at 165˚C for 120 s, (K) heat sealed SRDD device/catheter junctions, (L) heat shrink tubing trimmed off leaving fully fabricated SRDD device. Scale bar = 10 mm. SRDD, soft robotic drug delivery device; TPU, thermoplastic polyurethane.


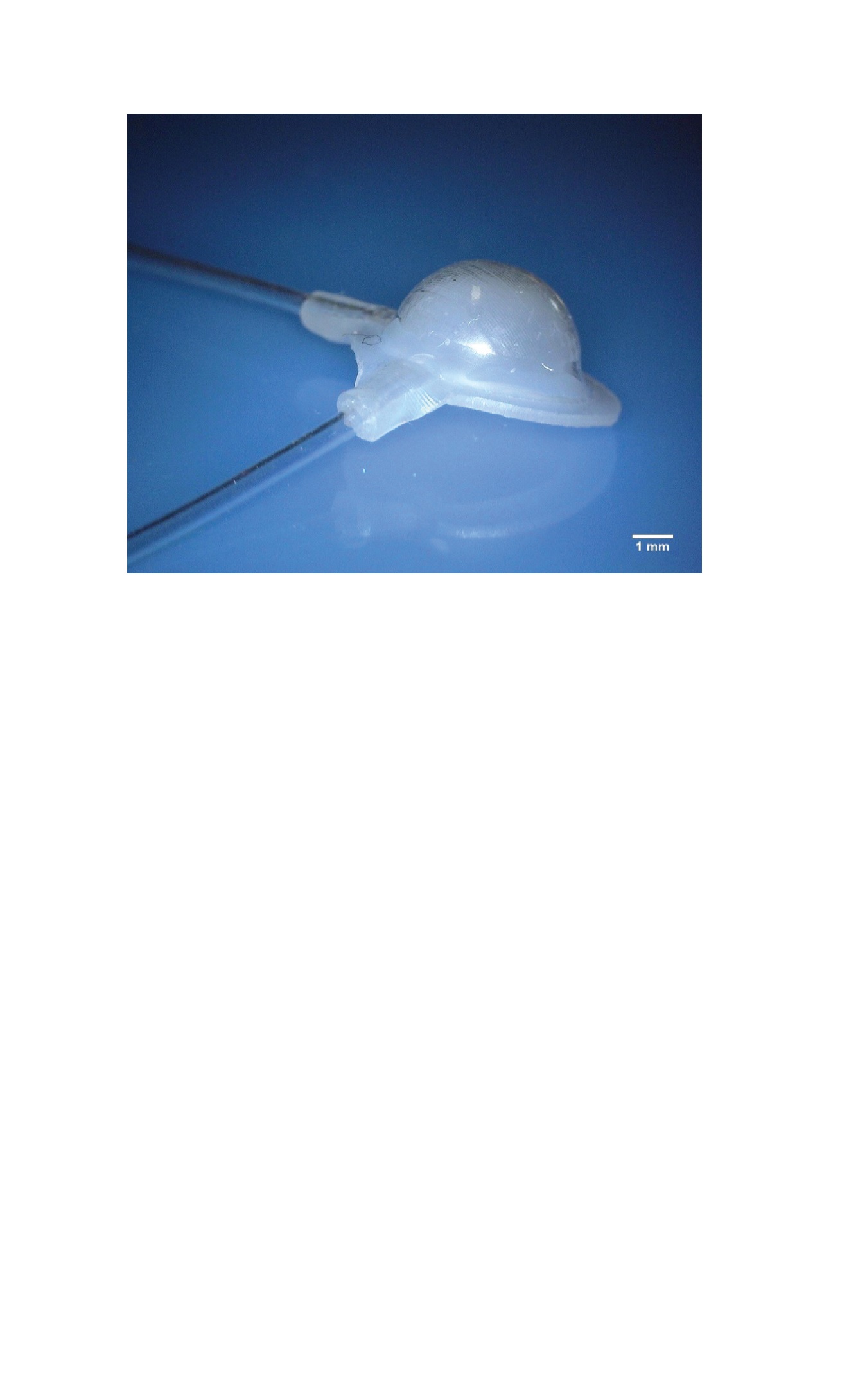


Figure S9 Rat scale SRDD device. SRDD, soft robotic drug delivery. Scale bar = 1 mm.


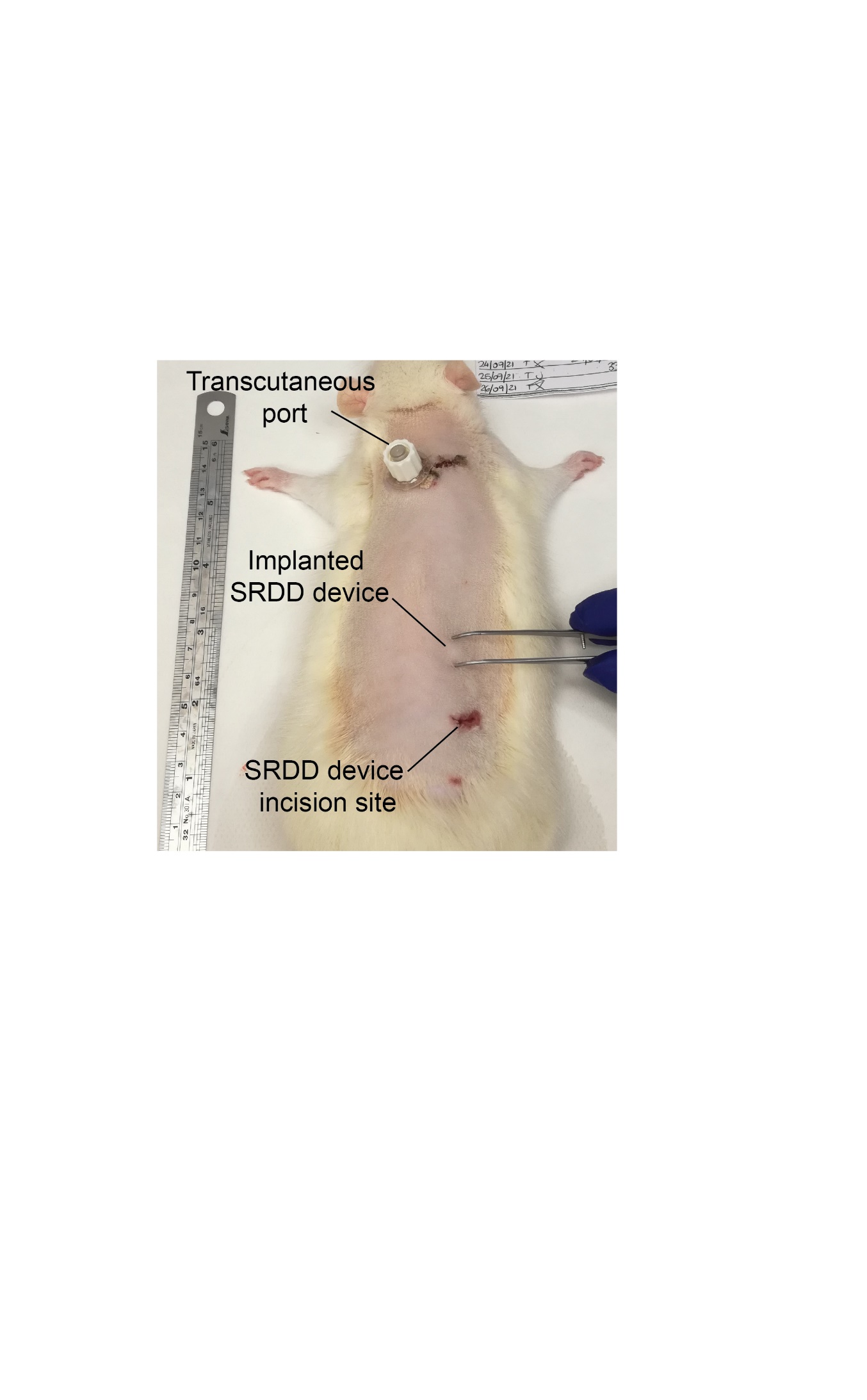


Figure S10 Healing of incision sites and position of implanted SRDD device and transcutaneous port on day 7 prior to euthanasia. SRDD, soft robotic drug delivery.

**Other supplementary files**

**Supplementary Movie 1** Injection of AA-CMC hydrogel through therapeutic catheter into therapeutic reservoir of SRDD device where it gels.

**Supplementary Movie 2** Actuation of SRDD device by custom-made electropneumatic control system via subcutaneous port.

**Supplementary Movie 3** Refilling of SRDD device with AA-CMC hydrogel after 70 consecutive actuations.
